# Unification of Opposites between Two Antioxidant Transcription Factors Nrf1 and Nrf2 in Mediating Distinct Cellular Responses to the Endoplasmic Reticulum Stressor Tunicamycin

**DOI:** 10.3390/antiox9010004

**Published:** 2019-12-19

**Authors:** Yu-ping Zhu, Ze Zheng, Shaofan Hu, Xufang Ru, Zhuo Fan, Lu Qiu, Yiguo Zhang

**Affiliations:** 1 The Laboratory of Cell Biochemistry and Topogenetic Regulation, College of Bioengineering and Faculty of Sciences, Chongqing University, No. 174 Shazheng Street, Shapingba District, Chongqing 400044, Chinazhengzeused@163.com (Z.Z.); hufan2441@163.com (S.H.); ruxufang@163.com (X.R.); 18623059592@163.com (Z.F.); qiulu@zzu.edu.cn (L.Q.); 2 School of Life Sciences, Zhengzhou University, No. 100 Kexue Avenue, Zhengzhou 450001, China

**Keywords:** Nrf1, Nrf2, antioxidant, ER stress, unfolded protein response (UPR), redox signaling, tunicamycin, proteasome, glycosylation, PERK, IRE1, ATF6, ATF4, HO-1, GCLM, topobiology

## Abstract

The water-soluble Nrf2 (nuclear factor, erythroid 2-like 2, also called Nfe2l2) is accepted as a master regulator of antioxidant responses to cellular stress, and it was also identified as a direct target of the endoplasmic reticulum (ER)-anchored PERK (protein kinase RNA-like endoplasmic reticulum kinase). However, the membrane-bound Nrf1 (nuclear factor, erythroid 2-like 1, also called Nfe2l1) response to ER stress remains elusive. Herein, we report a unity of opposites between these two antioxidant transcription factors, Nrf1 and Nrf2, in coordinating distinct cellular responses to the ER stressor tunicamycin (TU). The TU-inducible transcription of *Nrf1* and *Nrf2*, as well as *GCLM* (glutamate cysteine ligase modifier subunit) and *HO-1* (heme oxygenase 1), was accompanied by activation of ER stress signaling networks. Notably, the unfolded protein response (UPR) mediated by ATF6 (activating transcription factor 6), IRE1 (inositol requiring enzyme 1) and PERK was significantly suppressed by Nrf1α-specific knockout, but hyper-expression of Nrf2 and its target genes *GCLM* and *HO-1* has retained in *Nrf1α*^−/−^ cells. By contrast, *Nrf2*^−/−*ΔTA*^ cells with genomic deletion of its transactivation (TA) domain resulted in significant decreases of GCLM, HO-1 and Nrf1; this was accompanied by partial decreases of IRE1 and ATF6, rather than PERK, but with an increase of ATF4 (activating transcription factor 4). Interestingly, Nrf1 glycosylation and its *trans*-activity to mediate the transcriptional expression of the 26S proteasomal subunits, were repressed by TU. This inhibitory effect was enhanced by *Nrf1α*^−/−^ and *Nrf2*^−/−^*^ΔTA^*, but not by a constitutive activator *caNrf2^ΔN^* (that increased abundances of the non-glycosylated and processed Nrf1). Furthermore, *caNrf2^ΔN^* also enhanced induction of PERK and IRE1 by TU, but reduced expression of ATF4 and HO-1. Thus, it is inferred that such distinct roles of Nrf1 and Nrf2 are unified to maintain cell homeostasis by a series of coordinated ER-to-nuclear signaling responses to TU. Nrf1α (i.e., a full-length form) acts in a cell-autonomous manner to determine the transcription of most of UPR-target genes, albeit Nrf2 is also partially involved in this process. Consistently, transactivation of ARE (antioxidant response element)-driven *BIP* (binding immunoglobulin protein)-, *PERK*- and *XBP1* (X-box binding protein 1)-*Luc* reporter genes was mediated directly by Nrf1 and/or Nrf2. Interestingly, Nrf1α is more potent than Nrf2 at mediating the cytoprotective responses against the cytotoxicity of TU alone or plus tBHQ (*tert*-butylhydroquinone). This is also further supported by the evidence that the intracellular reactive oxygen species (ROS) levels are increased in *Nrf1α*^−/−^ cells, but rather are, to our surprise, decreased in *Nrf2*^−/−*ΔTA*^ cells.

## 1. Introduction

**Figure 1:**
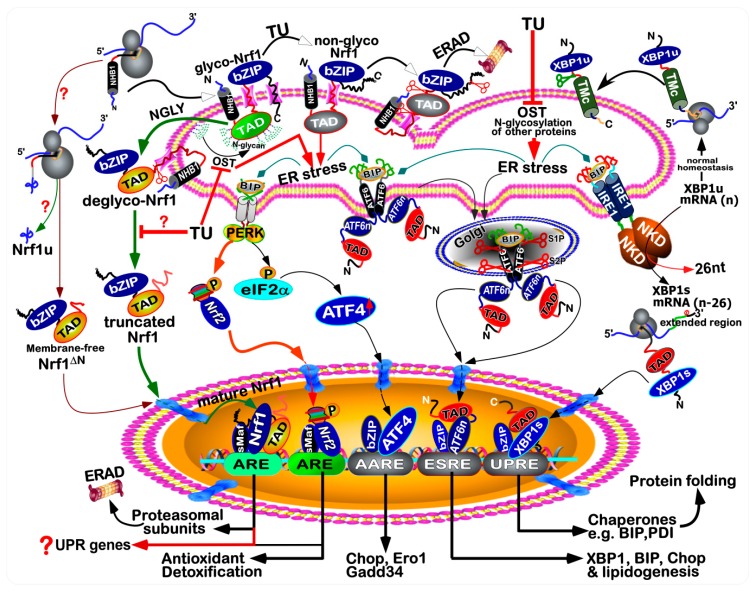
Inhibition of Nrf1 N-glycosylation by tunicamycin (TU) in the endoplasmic reticulum (ER)- to-nuclear signaling responses. A topobiological model is herein proposed to explain the inhibition of N-glycosylation of Nrf1 by TU, serving as a classic ER stressor, which can also block the ensuing deglycosylation and proteolytic processing of this cap’n’collar (CNC)-basic-region leucine zipper (bZIP) protein to yield a mature transcription factor. The resulting non-glycosylated Nrf1 and others are subjected to the ER-associated degradation (ERAD) pathway mediated by proteasomes. To the contrary, the non-glycosylated and unfolded proteins are accumulated insomuch as to stimulate ER stress. Thereby, inhibition of Nrf1 by TU is accompanied by the activation of a canonic ER-to-nuclear response signaling mediated by PERK, IRE1 and ATF6, three key transducers tethered to ER membranes as reviewed by the authors [1,2,3,4]. Of note, PERK was identified as a direct upstream kinase of Nrf2, as well as elF2α [6], but Nrf2 is neither translocated nor N-glycosylated in the lumen of ER. As such, Nrf2 is generally accepted as a master transcription factor to regulate cognate genes driven by antioxidant response elements (ARE) in the promoter regions. In such responses, ATF4 is selectively translated by the elF2α-controlled initiation machinery to regulate target genes (including *Chop*, *Ero1*, *Gadd34*) containing amino acid response elements (AARE) in their promoters. To get rid of ER stress, ATF6 is activated by its consecutive proteolytic processing by Site-1 and Site-2 proteases (i.e., S1P and S2P, respectively) in the Golgi apparatus. The full-length mRNA of XBP is alternatively spliced to remove its 26 nucleotides at the nearly 3′-end, such that the subsequent portion of its open reading frame is shifted so much as to yield another inducible protein isoform, called XBP-1s, that is longer in size than its original protein of XBP-1u. Consequently, distinct subsets of cognate genes containing *cis*-regulatory ESRE (ER stress response element) or UPRE (unfolded protein response element) are regulated transcriptionally by ATF6n (i.e., its active N-terminal portion) and XBP-1s, respectively. In addition, a putative micropeptide Nrf1u is likely generated by a translation of the upstream open reading frame existing in the full-length mRNA transcript of this CNC-bZIP factor, particularly under ER stress conditions. Certainly activated or blocked nodes in the some cascades are denoted by arrows (→) or bars (⊥), respectively. Scissors represent putative protein processing

As a highly-evolved organelle of eukaryotic cells, endoplasmic reticulum (ER) is of crucial importance to be involved in the biosynthesis of secretory and membrane proteins, as well as lipids including cholesterol, proper folding of proteins and specific post-translational modifications (e.g., N-glycosylation) of those proteins sorted out of the ER to their destination organelles. The ability of the ER to orchestrate intracellular proteins and lipids is also severely challenged by a vast variety of physiopathological stresses, including environmental stimuli [1]. Consequently, disruption of such function of ER leads to accumulation of a large amount of unfolded and/or misfolded proteins in the lumen of this organelle, destroying the normal original homeostasis of the cell. This is known as ER stress to activate the unfolded protein response (UPR) [2]. In metazoans, the UPR is mediated principally by three major axes of signaling pathways from: (i) PERK-eIF2α (eukaryotic translation initiation factor 2α) to ATF4 and Chop (C/EBP homologous protein, also called DDIT3); (ii) IRE1 to XBP1; (iii) ATF6 [3]. In early stages of UPR, those unfolded proteins are allowed for binding to an ER luminal-resident chaperone BIP, also called GRP78 (glucose-regulated protein 78) [4]. This critical event results in the induction of those ER-associated sensors PERK, IRE1 and ATF6 (as illustrated in Figure 1), so that their relevant UPR signaling pathways are subsequently activated [5].

Upon activation of UPR, it is also accompanied by concomitant and secondary induction of a complex signaling network; this includes pro-survival mechanisms involving antioxidant responses, ER-associated degradation (ERAD), ER biogenesis and autophagy [7]. Notably, the potential role of UPR in maintaining redox homeostasis is attracting the great interest of workers in different fields [8]. Clearly, it is generally accepted that redox signaling responses are mediated primarily by two important antioxidant transcription factors, Nrf1 and Nrf2 [9]. These two major members of the cap’n’collar (CNC) basic-region leucine zipper (bZIP) family predominantly regulate transcription of antioxidant/electrophile response elements (AREs/EpREs)-driven genes involved in detoxification and other cytoprotective adaptation. Besides, Nrf2 was also previously reported to be significantly upregulated by the amyloid β-induced ER stress leading to UPR [10]. As one of those three UPR transducers, PERK had been identified to be a direct upstream kinase of Nrf2 [6], which is well characterized as a master antioxidant transcription factor to counterbalance the harmful effects of reactive oxygen species (ROS) in cells. Under ER stress, Nrf2 is phosphorylated by PERK, such that the phosphorylated Nrf2 dissociates from its negative regulator Keap1 (kelch like ECH associated protein 1) and then translocates the nucleus, leading to transactivation of ARE-battery genes [11,12]. In the meanwhile, eIF2α is phosphorylated by PERK, thereby repressing general protein translation, but promoting selective protein translation of ATF4 [13,14,15]. Furtherly, the heterodimer consisting of ATF4 and Nrf2 binds to the stress-response element of HO-1, before inducing this gene expression [16]. Collectively, these demonstrate that the Nrf2-mediated expression of ARE genes is activated in the UPR signaling to ER stress. However, whether (and how) the ER membrane-associated Nrf1- mediated response signaling is triggered to the accompaniment of UPR remains, to date elusive.

The ER-associated Nrf1 adapts a unique membrane-topology (as shown in Figure 1), at least in part, of which is locally similar to the consensus transmembrane topology of PERK, IRE1 and ATF6. Thereby, it is postulated that the membrane-bound Nrf1 should, theoretically, have a strong capacity of being induced by ER stress, as described for the homolog of *Caenorhabditis elegans* Skn-1 [17,18,19]. Intriguingly, ectopically-expressed Nrf1 protein appeared to be *de facto* not activated by each of these UPR signaling pathways, but conversely, activation of Nrf1 by *tert*-butylhydroquinone (tBHQ) or arsenic to up-regulate distinct ARE-driven genes responsible for antioxidant, detoxification and cytoprotection, was repressed by classic ER stressors, tunicamycin (TU), thapsigargin (TG) and brefeldin A (BFA) [20,21]. As such, both the electrophoretic mobility of Nrf1 and its subcellular redistribution were altered by TU and BFA, but not TG [21]. Thereby, this suggests that Nrf1 is modified in an ER stress-specific post-translational fashion (e.g., N-glycosylation, deglycosylation, ubiquitination and proteolysis). By contrast, the water-soluble Nrf2 was identified as a direct target of PERK triggered by TU, insofar as to activate antioxidant and detoxification responses [6,12]. 

It is also important to note a unique UPR-independent mechanism whereby the transactivation activity of Nrf1, but not Nrf2, to up-regulate transcriptional expression of all those genes encoding 26S proteasomal (*PSM*) subunits, is induced by the low concentrations of its inhibitors [22,23,24]. Such proteasomal inhibition also results in the accumulation of oxidative, ubiquitinated proteins, thereby triggering an ER stress response [24,25,26]. In fact, the ER stress-inducible UPR signaling pathways were endogenously activated by loss of the mouse Nrf1’s function in the homozygous (*Nrf1*^−/−^) hepatocytes [27]. Contrarily, similar ER stress responses and resultant steatosis were enhanced by the half loss of Nrf1 in the heterozygous (*Nrf1*^+/−^) livers, when compared with wild-type (*Nrf1*^+/+^) livers, in response to 26S proteasomal inhibition [27]. The concurrence of severe oxidative stress with UPR in mouse *Nrf1*^−/−^ livers is resulted from down-regulation of antioxidant, detoxification and proteasomal genes. In the proteasomal compensatory response to limited extents of proteasome inhibitors [28], Nrf1 is subject to the multistep processing of this CNC-bZIP protein and its nuclear translocation [29], albeit the inhibition of proteasome-mediated ERAD is one of the causes of ER stress [24,26,30]. These facts demonstrate that Nrf1 should possess an essential cytoprotective role against the development of hepatosteatosis by maintaining the intracellular ER homeostasis. This is, in part, supported by the demonstration that Nrf1 is of crucial important for redox balance and the survival of mouse liver cells during development [31]. Thereby, Nrf1 is considered to mediate the basic cytoprotective response against oxidative stress in the pathogenesis of chronic liver diseases, e.g., nonalcoholic steatohepatitis (NASH) and its malignant transformation to liver cancer [32,33]. Such being the case, the mechanism underlying such integrative responses mediated by both Nrf1 and Nrf2 remains unknown, to date.

To unveil the mystery players of Nrf1 and Nrf2 in UPR, it is necessary to gain insights into the distinct roles of both CNC-bZIP factors in the ER signaling response to TU. Herein, we report a unity of the opposite roles of Nrf1 and Nrf2 in distinct cellular responses to treatment of TU in different genotypic cell lines. Such the TU-induced UPR signaling pathways by ATF6, IRE1 and PERK were significantly suppressed by Nrf1α-specific knockout, but Nrf2 along with GCLM and HO-1 were highly expressed in *Nrf1α*^−/−^ cells. By striking contrast, *Nrf2*^−/−*ΔTA*^ cells with genomic deletion of its transactivation (TA) domain resulted in significant decreases of GCLM, HO-1 and Nrf1. This was also accompanied by partial decreases of IRE1 and ATF6, but not PERK, along with an increase of ATF4. Notably, glycosylation of Nrf1 and its *trans*-ability to activate the transcriptional expression of 26S proteasomal subunits, were markedly repressed by TU. This inhibitory effect was enhanced by *Nrf1α*^−/−^ and *Nrf2*^−/−*ΔTA*^, but not by a constitutive activator *caNrf2^ΔN^* (because it increased abundances of non-glycosylated and processed Nrf1). Also, *caNrf2^ΔN^* enhanced the induction of PERK and IRE1 by TU, but reduced ATF4 and HO-1. Collectively, these distinctive roles of Nrf1 and Nrf2 in the ER-to-nuclear signaling responses to TU are integrally unified to maintain cell homeostasis. Overall, our results presented herein demonstrate that Nrf1α acts as a dominant player in a cell-autonomous manner to regulate most of the UPR genes expression, while Nrf2 is also involved in this process partially by IRE1, at least in this experimental setting. Consistently, our evidence also demonstrates that transactivation of luciferase reporter genes driven by ARE sequences from the *BIP*, *PERK* and *XBP1* promoter regions was mediated by Nrf1 and/or Nrf2. Intriguingly, Nrf1α is more potent than Nrf2 at mediating the cytoprotective response to the cytotoxic effects of TU alone or plus tBHQ. This notion is further supported by the surprising observations, showing that the intracellular ROS levels are elevated in *Nrf1α*^−/−^ cells, but rather suppressed in *Nrf2*^−/−*ΔTA*^ cells.

## 2. Materials and Methods

### 2.1. Cell Lines and Reagents

The human hepatocellular carcinoma HepG2 cells (i.e., *Nrf1/2*^+/+^) were obtained originally from the American Type Culture Collection (ATCC, Manassas, VA, USA). Three derived cell lines with knockout of *Nrf1α*^−/−^ or *Nrf2*^−/−*ΔTA*^ and constitutive activation of Nrf2 (i.e., *caNrf2^ΔN^*) were established in our laboratory; relevant characterization had been described in our previous publication by Qiu et al. [34]. Notably, the fidelity of HepG2 cell line had been conformed to be true by its authentication profiling and STR (short tandem repeat) typing map (which was carried out by Shanghai Biowing Applied Biotechnology Co., Ltd, Shanghai, China). They were cultured in a 37 °C incubator with 5% CO_2_, and allowed for growth in Dulbecco’s modified Eagle’s medium (DMEM) with 25 mmol/L high glucose, 10% (*v*/*v*) fetal bovine serum (FBS), 100 units/mL penicillin-streptomycin. 

The chemical TU (with MW 816.89) was purchased from Sangon Biotech Co., Ltd. (Shanghai, China). The antibody against Nrf1 was made in our own laboratory. All other five antibodies against Nrf2 (ab62352), GCLM (ab126704), HO-1 (ab52947) and XBP1 (ab109221) were purchased from Abcam (Cambridge, UK). Additionally, four antibodies against BIP (bs-1219R), Chop (bs-20669R), elF2α (bs-3613R) and p-IRE1 (bs-16698R) were from Bioss (Beijing, China), in addition to p-elF2α (#5199) from CST (Boston, USA, p-PERK (sc-32577) from (Santa Cruz, CA, USA), PSMB6 (A4053) from ABclonal (Wuhan, China) and β-actin (TA-09) from ZSGB-BIO (Beijing, China). 

### 2.2. Cell Viability and Cytoprotective Analysis

All four cell lines *Nrf1/2*^+/+^, *Nrf1α*^−/−^, *Nrf2*^−/−*ΔTA*^ and *caNrf2^ΔN^* were cultured for 24 h in DMEM containing 25 mmol/L glucose and 10% FBS. After reaching 70% of their confluence, they were then allowed for growth in fresh media containing different concentrations of TU (at 0, 0.5, 1, 2, 4 or 8 μg/mL), which was dissolved in DMSO (dimethyl sulfoxide; 0.1% of this solvent was herein used as a vehicle control). For their time-course, experimental cells were also treated with 2 μg/mL of TU for different lengths of time (i.e., 0, 4, 8, 12, 16, 20, or 24 h). The cell viability was then evaluated by using an MTT-based cell proliferation and cytotoxicity assay kit (Beyotime, Shanghai, China). 

For cytoprotective analysis, after these four cell lines reached 70% of their confluence, they were firstly allowed for 16-h growth in fresh media containing 50 μmol/L *tert*-butylhydroquinone (+ tBHQ, dissolved in DMSO) or without this redox inducer (−tBHQ) in 0.1% the vehicle DMSO. Then, these cells were or were not treated with 2 μg/mL TU for 0, 16, 24, 48 or 72 h, before being harvested. Subsequently, the cell survival and death were estimated by using the MTT-based cell proliferation and cytotoxicity assays. The data were shown as the percent changes (mean ± standard deviation (SD), n = 6), relative to the starting values measured from each of cell lines that had been treated with the vehicle DMSO or the indicated chemicals for 0 h (i.e., *T*_0_). These results are representative of at least three independent experiments, each of which was performed in quintuplicate.

### 2.3. The Constitutive Expression of the ER Stress-Related Genes in Selected Cell Lines

Equal numbers of *Nrf1/2*^+/+^, *Nrf1α*^−/−^, *Nrf2*^−/−*ΔTA*^ and *caNrf2^ΔN^* were cultured in 6-well plates before being harvested in a lysis buffer [35]. Total cell lysates were subjected to protein separation by SDS-PAGE gels containing 8–10% polyacrylamide, followed by Western blotting with antibodies against Nrf1 (made in our laboratory) and Nrf2 (from ABCAM, Cambridge, UK) or β-Actin (from Zhong Shan Jin Qiao Co., Beijing, China). β-Actin served as an internal control to verify the amounts of proteins that were loaded in each of the wells. Meantime, a portion of the differential expression genes were identified by transcriptome sequencing, and their relative basal expression levels were also calculated and presented as fold changes (mean ± SD) in the Reads Per Kilobase per Million mapped reads (RPKM). According to the Log2-based RPKM values against those determined from *Nrf1/2*^+/+^, the heatmaps for experimented cell lines were generated by using the MEV4.9.0 program (Dana-Farber Cancer Institute, Boston, MA, USA). 

### 2.4. The mRNA Expression of the Examined Responsive Genes to TU

After reaching 70% confluence of *Nrf1/2*^+/+^, *Nrf1α*^−/−^, *Nrf2*^−/−*ΔTA*^ and *caNrf2^ΔN^* cell lines grown in DMEM containing 25 mmol/L glucose and 10% FBS, they were treated for different time periods with 2 μg/mL of TU. Their total RNAs were extracted by using an RNA extraction kit (TIANGEN, Beijing, China), 500 ng of which was then subjected to the reactions with reverse transcriptase (Promega, Madison, WI, USA) to synthesize the single strand cDNAs, that served as PCR templates. Subsequently, relative mRNA expression levels of both ER stress-related and proteasomal genes in these experimental cell lines were measured by qRT-PCR with each of the indicated pairs of primers (as listed below in Table 1).

This reaction was carried out in the GoTaq^®^ real-time PCR detection systems, loaded on a CFX96 instrument (Bio-rad, Hercules, CA, USA), before being deactivated at 95 °C for 10 min, and amplified by 40 reaction cycles of 15 s at 95 °C and 30 s at 60 °C. The resulting data were analyzed by the Bio-Rad CFX Manager 3.0 software (Hercules, CA, USA). The final melting curve was validated to examine the amplification quality, whereas the mRNA f expression of β-actin was here viewed as an internal standard control (because it is the most stable housekeeping gene selected from multiple housekeeping genes). All the resulting data were shown as fold changes (mean ± SD, n = 6), relative to the basal values obtained from *Nrf1/2*^+/+^ cells that had been treated with the vehicle DMSO or the indicated chemicals for 0 h (i.e., *T*_0_).

### 2.5. The Protein Expression of the Examined Responsive Genes to TU

After reaching 70% confluence of *Nrf1/2*^+/+^, *Nrf1α*^−/−^, *Nrf2*^−/−*ΔTA*^ and *caNrf2^ΔN^* cell lines growth in DMEM containing 25 mmol/L glucose and 10% FBS, they were treated with TU (2 μg/mL) for distinct lengths of time from 0 to 24 h. Their total lysates were separated by SDS-PAGE gels containing 10% polyacrylamide, followed by Western blotting with distinct primary antibodies against GCLM, BIP, Chop, eIF2α, HO-1, p-IRE1, XBP1 and PSMB6. The β-Actin served as an internal control to verify the amounts of proteins that were loaded into each of the wells. The intensity of some immunoblots was quantified by the Quantity One 4.5.2 software (Bio-rad, Hercules, CA, USA) and shown graphically. All of the indicated immunoblots were firstly normalized to their loading control β-Actin intensity, and then calculated as fold changes (mean ± SD, n = 3), relative to the basal levels obtained from each of cell lines that had been treated with the vehicle controls or the indicated chemicals for 0 h (i.e., *T*_0_).

### 2.6. Flow Cytometry Analysis of Cell Apoptosis and Intracellular ROS Levels

Equal numbers (3 × 10^5^) of experimental cells *Nrf1/2*^+/+^, *Nrf1α*^−/−^, *Nrf2*^−/−*ΔTA*^ and *caNrf2^ΔN^* were seeded into each well of 6-well plates. After reaching 70% of their confluence, the cells were allowed for growth in fresh media containing tBHQ (50 μmol/L) or the vehicle DMSO (i.e., −tBHQ) for 16 h, before they were or were not treated with 2 μg/mL of TU for an additional 48 h. The cells were subsequently incubated for 20 min at 37 °C in a serum-free medium containing 10 μmol/L of 2′,7′-dichlorodihydrofluorescein diacetate (DCFH-DA) (Beyotime, Shanghai, China). The cells were rinsed for three times in serum-free media, followed by flow cytometry analysis of the intracellular green fluorescent intensity (representing the ROS level). A similar procedure was also subjected to apoptosis analysis of the above-mentioned four cell lines. These cells were pelleted by centrifuging at 1000× *g* for 5 min, and then washed in PBS for three times, before being incubated in 195 μL of a binding buffer containing 5 μL of Annexin V-FITC and 10 μL of propidium iodide (PI) for 15 min, followed by flow cytometry analysis of cell apoptosis. The resulting data were further analyzed by the FlowJo 7.6.1 software (FlowJo, Ashland, OR, USA).

### 2.7. Luciferase Reporter Assays of ARE-Driven Gene Trans-Activity

Experimental cells (1.5 × 10^5^) were seeded into each well of the 12-well plates. After reaching 80% confluence, the cells were co-transfected using a Lipofectamine 3000 mixture with each of the *ARE*-driven luciferase plasmids (which were made by inserting each of the indicated ARE sequences into the pGL3-Promoter vector) or non-ARE reporter plasmids (as a background control), together with an expression construct for Nrf1, Nrf2 or empty pcDNA3.1 vector. In this system, the *Renilla* expression by pRL-TK plasmid served as an internal control for transfection efficiency. In addition, the Pyralis-luciferase activity in the psi-CHECK2 plasmid system was, sometimes, also viewed as an internal control. The luciferase activity was measured by the dual-luciferase reporter system (Beyotime, Shanghai, China). These resultant data were firstly normalized to their corresponding backgrounds obtained from co-transfection of cells with non-ARE reporter and relevant expression constructs, and then calculated as a fold change (mean ± SD, n = 6) relative to the activity of the basal levels (at a given value of 1.0) obtained from transfection of cells with the empty pcDNA3.1 vector and each of the indicated *ARE*-driven luciferase plasmids. All the data presented in this study represent at least three independent experiments undertaken on separate occasions that were each performed in triplicate. Significant differences in the ARE-driven transactivity mediated by Nrf1 and/or Nrf2 were subjected to statistical analysis, when compared to the basal values.

### 2.8. Statistical Analysis

The ’wet’ experimental data provided in this study were represented as the mean ± SD, and were analyzed using the Student’s *t*-test or Fisher’s exact test, as appropriate. The resulting value of *p* < 0.05 was considered as a significant difference. In addition, statistical determination of the ’dry’ sequencing analysis was described by Wang et al. [36]. 

## 3. Results

### 3.1. Both Nrf1α and Nrf2 Contribute to Differential Expression of Responsive Genes to the Basal ER Stress in Four Different Genotypic Cell Lines

**Figure 2:**
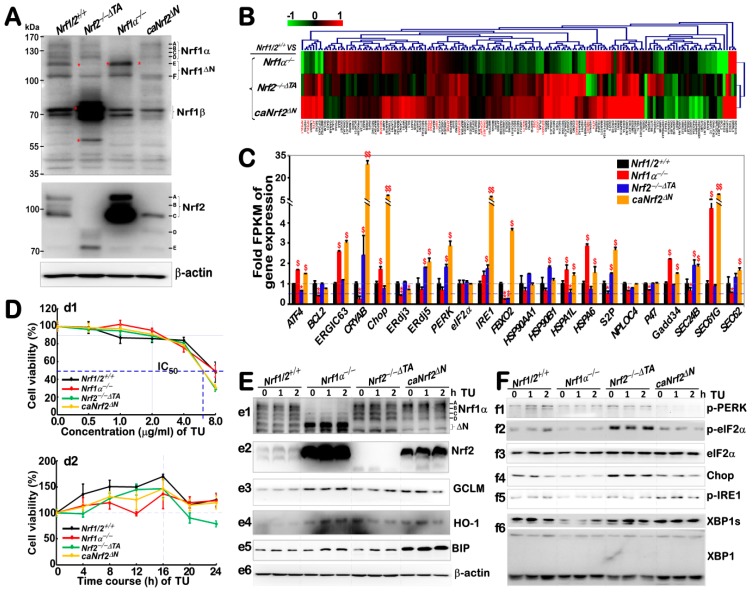
Distinct contributions of Nrf1α and Nrf2 to the differential expression of ER stress-related genes. (**A**) Distinct protein levels of Nrf1 and Nrf2 in four different genotypic cell lines *Nrf1/2*^+/+^, *Nrf1α*^−/−^, *Nrf2*^−/−*ΔTA*^ and *caNrf2^ΔN^* were determined by Western blotting with specific antibodies. (**B**) A heatmap was made by the Log2-based RPKM (Reads Per Kilobase per Million mapped reads) values, representing differential expression profiles of those ER stress-responsive genes, by comparison to those obtained from *Nrf1*^+/+^ cells. Changes in basal expression of these genes are shown, to distinct degrees of colors. The enlarged images with a higher resolution are shown in Supplemental Figure S1. (**C**) The expression levels of ER stress-responsive genes were also evaluated as fold changes in the RPKM values. Significant statistical decreases were indicated by *p< 0.01 or **p < 0.001, whereas significant increases were represented by *$*, *p* < 0.01 or *$$*, *p* < 0.001. (**D**) Cell viability was determined by the MTT-based assay, after these cell lines had been treated with different concentrations of TU for 24 h (*d1*) or treated with 2 μg/mL of TU for different lengths of time (*d2*). (**E**) The protein levels of antioxidant genes (i.e., *e1 to e6*) and (**F**) the ER stress-related genes (i.e., *f1 to f6*) in these experimental cell lines, that had been treated only with 2 μg/mL TU for a short time (from 0 to 2 h), were examined by Western blotting with different primary antibodies as indicated. It is important to note that these cell lines were not pretreated in the routine, complete culture medium, before being experimented.

To explore the distinct functions of Nrf1 and Nrf2 in the putative ER stress response, herein we employed four different genotypic cell lines, which had been established by gene-editing with the presence or absence of Nrf1α and Nrf2 (for detailed characterization of these cell lines, please peruse this publication [34]). Consistently, these selected cell lines were re-identified by Western blotting of Nrf1 and Nrf2 before RNA sequencing. As shown in Figure 2A, the wild-type HepG2 cell line (*Nrf1/2*^+/+^) served as a control for the expression of Nrf1 and Nrf2. By contrast, the *Nrf1α*^−/−^ cell line exhibited a clear disappearance of intact, full-length Nrf1α/TCF11 protein and its derivatives (which are embodied by glycoprotein-A, deglycoprotein-B and their N-terminally-truncated C-/D-forms) of between ~140-kDa and ~120 kDa; their detailed identifications had been described in our previous work [24,37]. However, expression of the shorter truncated E-form of Nrf1 appeared to be enhanced in *Nrf1α*^−/−^ cell, but with the unaltered F-form, when compared with *Nrf1/2*^+/+^ cells (Figure 2A, *upper panel*). This implies a potential molecular compensatory mechanism, because both E- and F-forms of Nrf1 may be also generated by the translation of a shorter-length, open reading frame of mRNA resulting from alternative splicing to move the first exon (i.e., Nrf1^ΔN^) [9,24], in addition to the proteolytic processing of the longer Nrf1 isoforms to yield its mature factor. Intriguingly, almost no expression of the Nrf1-truncated E-isoform was determined in both *Nrf2*^−/−*ΔTA*^ and *caNrf2^ΔN^* cell lines, but they gave a modest decrease in abundance of the Nrf1 F-form. Similarly, abundances of Nrf1β bands close to 70 kDa appeared to be unaffected by the knockout of Nrf1α, but was significantly augmented by *Nrf2*^−/−*ΔTA*^, but rather evidently reduced by *caNrf2^ΔN^* (Figure 2A, *upper panel*). These data suggest a potential effect of Nrf2 on the alternative translation of either Nrf1^ΔN^ or Nrf1β, but another possible role of Nrf2 in the alternative transcription of Nrf1 cannot also be ruled out, albeit any detailed mechanism(s) remains unknown. 

By contrast with *Nrf1/2*^+/+^ cells, the *Nrf1α*^−/−^ cells gave rise to a dramatic increase in the expression of Nrf2 between ~100-kDa and 110-kDa (Figure 2A, *middle panel*); such a demonstrating effect of Nrf1 on the expression of Nrf2 was also described previously [34]. Nonetheless, similar longer Nrf2 of ~100-110 kDa were completely abolished in *Nrf2*^−/−*ΔTA*^ cells, but replaced by additional smaller molecular weight polypeptides with genomic loss of its transactivation domains (i.e., Neh4 and Neh5). Notably, the intact, full-length Nrf2 of ~110-kDa was also totally abolished by *caNrf2^ΔN^*, but it still retained a major short Nrf2 of ~100 kDa, together with a few of minor small polypeptides (Figure 2A, *middle panel*). These suggest that possible proteolytic processing of Nrf2 may occur within its N-terminal Keap1-binding Neh2 domain (which is highly conserved with the Neh2L region of Nrf1, immediately adjacent to its N-terminal domain targeting to the ER).

Subsequently, the fidelity of total RNAs purified from the above-identified four cell lines was rigidly confirmed to be available for RNA-sequencing. A heatmap of the sequencing data revealed 157 of the differentially expressed genes clustered responsibly for basal ER stress in *Nrf1α*^−/−^, *Nrf2*^−/−*ΔTA*^ or *caNrf2^ΔN^* by comparison with *Nrf1/2*^+/+^ cells (Figure 2B). Amongst them, relative highly expressed genes differed in these four cell lines as shown graphically (Figure 2C). Further analysis of these data, together with the aforementioned alternations in abundances of Nrf1 and Nrf2, suggests that basal expression of seven genes, such as *ATF4*, *Chop*, *Gadd34*, *ERGIC63*, *HSPA1L*, *HSPA6*, and *Sec61γ* (encoding a component of Sec61 ER-translocon complex) should be predominantly regulated by Nrf2, because their mRNA expression levels were significantly increased in *Nrf1α*^−/−^ and *caNrf2^ΔN^* cells, but obviously diminished or even roughly abolished in *Nrf2*^−/−*ΔTA*^ cells (Figure 2C). By contrast, Nrf1α/TCF11 may be primarily involved in regulating the basal expression of an additional nine genes, namely, *BCL2*, *CRYAB*, *ERdj3*, *PERK*, *FBXO2*, *S2P*, *NPLOC4*, *Sec24β* and *Sec62* (of note, the latter two genes are responsible for protein transport and sorting out of the ER to their destinations). This is due to the fact that their basal mRNA abundances were markedly repressed in *Nrf1α*^−/−^ cells, albeit with high expressions of Nrf2. Conversely, a few of these genes regulated by Nrf1α might also be inhibited by Nrf2, because their mRNA expression levels were strikingly recovered by *Nrf2*^−/−*ΔTA*^ (though lacking most of its transactivation domains) or by *caNrf2^ΔN^* (albeit with a constitutive loss of the cytoplasmic Keap1-binding Neh2 domain of Nrf2, implying an unidentified nuclear function exerted by the Neh2 domain). Notably, IRE1 could also be co-regulated by Nrf1α and Nrf2, because its mRNA expression levels were unaffected by the knockout of either Nrf1 or Nrf2, but significantly elevated by *caNrf2^ΔN^*, implying a possible release of inhibition by Neh2.

### 3.2. Distinctive Effects of Nrf1α and Nrf2 on Basal Expression of Antioxidant and UPR-Related Proteins, and Their Responsive Expression at the Early Stages of TU-Stimulated ER Stress

To determine effects of Nrf1 and Nrf2 on the differential expression of putative genes in response to the ER stressor TU, we performed Western blotting to examine changes in the protein levels of early TU-responsive genes expressed in four different cell lines with the presence or absence of Nrf1α and Nrf2. Firstly, the cytotoxicity of TU was evaluated to provide an optimal concentration of this chemical that was treated in cells for an optimal time course (Figure 2D). A half of the maximal inhibitory concentration (IC_50_) of TU treated in *Nrf2*^−/−*ΔTA*^ or *caNrf2^ΔN^* cells was ~6.5 μg/mL, while another IC_50_ of the TU treatment of *Nrf1α*^−/−^ or *Nrf1/2*^+/+^ cells was close to 8.0 μg/mL (Figure 2, *d1*). Of note, 2.0 μg/mL of TU showed almost no obvious cytotoxicity within 24 h (Figure 2, *d2*), and thus was selected for the use of our subsequent experiments.

Western blotting revealed a modest increase in abundances of the longer inactive Nrf1 isoforms A/B; they were examined following 2-h TU-treatment of *Nrf1/2*^+/+^ or *caNrf2^ΔN^*, but not of *Nrf2*^−/−*ΔTA*^ cells (Figure 2E, *e1*). This appeared consistent with our previous work [24,38]. As such, two shorter active isoforms-C/D of Nrf1 were enhanced by the TU treatment of *Nrf2*^−/−*ΔTA*^ cells only. Notably, Nrf2 protein levels were obviously promoted following the TU treatment of *Nrf1α*^−/−^ or *caNrf2^ΔN^* cells (Figure 2E, *e2*). Further examination unraveled that the protein expression of ARE-driven genes encoding GCLM and HO-1 (both regulated by Nrf1 and/or Nrf2) was significantly increased by TU treatment of *Nrf1α*^−/−^ and *caNrf2^ΔN^* cells, when compared with *Nrf1/2*^+/+^ cells (Figure 2E, *e3* and *e4*). Conversely, this antioxidant effect was partially reduced in TU-treated *Nrf2*^−/−*ΔTA*^ cells. Together, these demonstrate that the short-term stimulation of TU can also trigger the induction of antioxidant response genes mediated by Nrf1 and Nrf2. 

Furtherly, different extents of increases in the chaperone BIP (also called GRP78, as a landscape signature of the classic TU-induced ER stress response) were determined following TU treatment of *caNrf2^ΔN^*, *Nrf1α*^−/−^, *Nrf2*^−/−*ΔTA*^ or *Nrf1/2*^+/+^ cells, when compared with their untreated controls (Figure 2E, *e5*). Relatively, higher expression levels of basal and TU-stimulated BIP were found in *caNrf2^ΔN^* cells (also with increased Nrf1). By contrast, significant elevation of Nrf2 from in *Nrf1α*^−/−^ cells only gave rise to a considerable level of BIP, but was also slightly suppressed by inactivation of Nrf2 in *Nrf2*^−/−*ΔTA*^ cells to a similar low level to that obtained from *Nrf1/2*^+/+^ cells. These data suggest that both Nrf1 and Nrf2 are required for basal expression of BIP and its inducible increment in a short-term ER stress response to TU. 

Western blotting examination of the PERK-eIF2α/Nrf2-Chop signaling pathway revealed that phosphorylated PERK and eIF2α proteins were modestly stimulated by short-term TU treatment of *Nrf1/2*^+/+^ cells (Figure 2F, *f1* and *f2*), but almost no changes in total Chop were observed within 1–2 h of TU treatment (*f4*). Interestingly, their expression was obviously attenuated by hyper-expression of Nrf2 in *Nrf1α*^−/−^ or *caNrf2^ΔN^* cells, but significantly recovered by *Nrf2*^−/−*ΔTA*^ to considerable high levels relative to those obtained from *Nrf1/2*^+/+^ cells; such alterations appeared to be independent of stress stimulated by TU. No matter what it is, these observations demonstrate that Nrf1 and Nrf2 have exerted opposing roles for the PERK-eIF2α-Chop signaling in different cellular response to a short- term stimulation of TU. Further examination of the IRE1-XBP1 pathway unraveled that basal (and short-term, TU-stimulated) expression of p-IRE and XBP1s appeared to be reduced in *Nrf1α*^−/−^ cells (Figure 2F, *f5* and *f6*), but p-IRE1 expression was enhanced in *caNrf2^ΔN^* cells, albeit no changes of both proteins were observed in *Nrf2*^−/−*ΔTA*^, by comparison with the equivalents of *Nrf1/2*^+/+^ cells. Together, these results suggest that Nrf1α, but not Nrf2, is required for an expression of auto-phosphorylated IRE1 and its target product XBP1s. However, it is hard to reach a conclusion from rather minimal or no effects of short-term TU exposure on the above-examined protein expression in *Nrf1/2*^+/+^ cells.

### 3.3. Nrf1α and Nrf2 Mediate Distinct Transcriptional Responses of Antioxidant and most UPR-Target Genes to the Long-Term TU-Stimulated ER Stress

**Figure 3:**
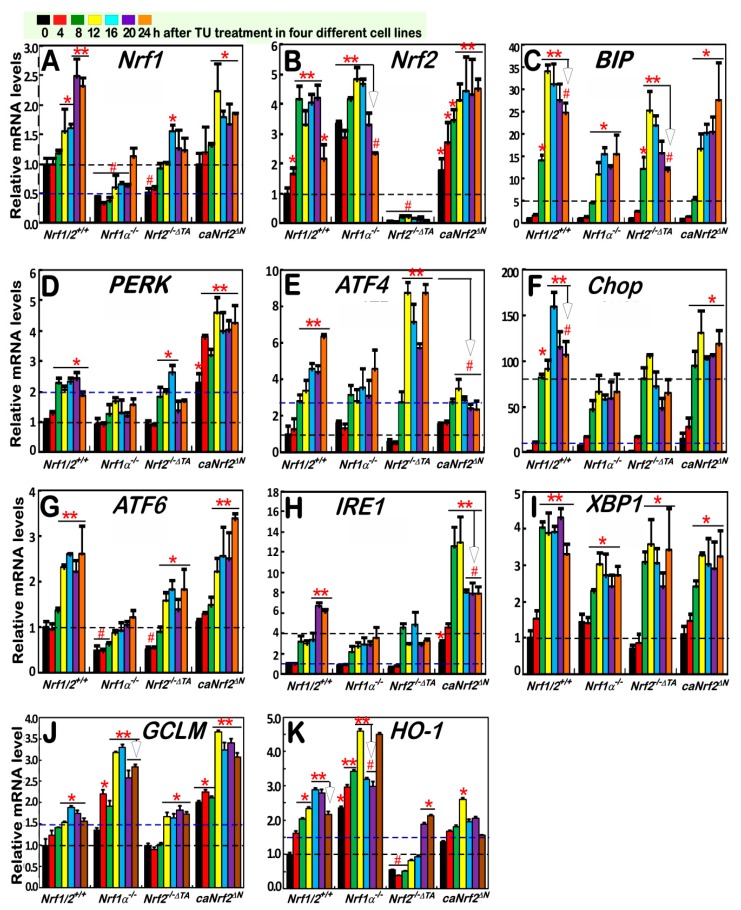
Time-dependent changes in the TU-inducible mRNA expression of distinct responsive genes. Distinct cell lines of *Nrf1/2*^+/+^, *Nrf1α*^−/−^, *Nrf2*^−/−*ΔTA*^ and *caNrf2^ΔN^* were or were not treated with 2 μg/mL of TU for the indicated times from 0 to 24 h. Subsequently, TU-inducible mRNA expression levels of the indicated genes were determined by real-time qPCR analysis. These examined genes included *Nrf1* (**A**), *Nrf2* (**B**), *BIP/GRP78* (**C**), *PERK* (**D**), *ATF4* (**E**), *Chop* (**F**), *ATF6* (**G**), *IRE1* (**H**), *XBP1* (**I**), *GCLM* (**J**) and *HO-1* (**K**). Significant statistical decreases were indicated with #, *p* < 0.01, whereas significant increases were represented by *p < 0.01 and **p < 0.001, respectively. In addition, it is noted that no significant differences between untreated (i.e., NC) and the vehicle (i.e., 0.1% dimethyl sulfoxide (DMSO)-treated mRNA expression levels of *BIP*, *HO-1*, *Nrf1* and *Nrf2* in wild-type HepG2 (*Nrf1/2*^+/+^) cells were also determined by qRT-PCR) (as shown in supplemental Figure S2).

The above experiments showed no or fewer changes in the inducible protein expression levels of some responsive genes at the early stages of TU-stimulated ER stress (Figure 2E,F). Of note, a few of these genes and their products were *de facto* auto-activated in a TU-independent fashion (e.g., autophosphorylation) under untreated homeostatic conditions. Therefore, the TU-treated time was further extended from within 4 h to 24 h, in order to determine distinctions in between Nrf1- and Nrf2-mediated transcriptional responses to a long-term, TU-stimulated ER stress. For this end, four different genotypic cell lines had been treated with TU for distinct lengths of time from 0 to 24 h, before these genotypic mRNAs were subjected to further analysis by real-time quantitative PCR (qRT-PCR). As anticipated, the results demonstrated that TU treatment of *Nrf1/2*^+/+^ cells triggered time-dependent increases in the transcriptional expression of *Nrf1* and *Nrf2* (Figure 3A,B). By contrast, basal and TU-stimulated mRNA expression levels of *Nrf1* were substantially abolished by *Nrf1α*^−/−^ (Figure 3A), albeit it retained residual shorter Nrf1 isoforms, as well as hyper-expression of Nrf2 (as shown in Figure 2A). Similarly, significant diminishments in the mRNA expression levels of *Nrf1* were also observed in *Nrf2*^−/−*ΔTA*^, but not *caNrf2^ΔN^* cells (Figure 3A). Together with the above-described data (Figure 2A), these further support our previous notion that transcription of the single *Nrf1* gene is regulated positively by itself factor Nrf1α (and its longer derivatives), as well as by Nrf2, as described by Qiu et al. [34], apart from a possible negative regulation by Nrf1β. 

Further qRT-PCR analysis of *Nrf1/2*^+/+^ cells revealed that a TU-stimulated increase in the mRNA expression of *BIP* (as a classic marker of UPR induced by ER stress) from 8 h to 24 h treatment, with a peak of TU induction, occurred at 12 h (Figure 3C). By striking contrast, such TU-inducible mRNA expression of *BIP* was substantially suppressed and also postponed in *Nrf1α*^−/−^ cells (albeit with hyper-expression of Nrf2). Also, *Nrf2*^−/−*ΔTA*^ cells only gave a modest reduction of TU-inducible *BIP* expression, with a lowered peak at 12 h and a subsequent downward course to 24 h after treatment. Collectively, these demonstrate that Nrf1 is required for transcriptional regulation of UPR-target *BIP* gene responsible for sensing ER stress, while Nrf2 is also partially involved in this response to TU. However, it is intriguing to note that *caNrf2^ΔN^* (acting as a constitutive CNC-bZIP activator) also caused a partial decrease of TU-inducible *BIP* expression (Figure 3C). This decrease is attributable to a loss of the Keap1-binding Neh2 domain from Nrf2 (to yield *caNrf2^ΔN^*), albeit a putative nuclear function of Neh2 is not yet identified to date.

Analysis of the PERK-eIF2α-ATF4-Chop response pathway unraveled the revelation that the TU treatment of *Nrf1/2*^+/+^ cells caused distinct time-dependent increases in the mRNA levels of *PERK*, *ATF4* and *Chop* from 8 h to 24 h (Figure 3D–F), which occurred with their respective peaks at 8 h, 24 h and 16 h, respectively. Of note, the TU-inducible expression of *PERK* was substantially reduced in *Nrf1α*^−/−^ cells (with high expression of Nrf2) but appeared to be almost unaffected by *Nrf2*^−/−*ΔTA*^, when compared with those obtained from *Nrf1/2*^+/+^ cells (Figure 3D). From these findings, it is postulated that Nrf1α, but not Nrf2, is essential for transcriptional regulation of *PERK* in response to TU. Nevertheless, *caNrf2^ΔN^* gave rise to rather significant increments in the basal and TU-induced mRNA expression of *PERK* (Figure 3D). Hence, it is inferable that, once Nrf2 is localized in the nucleus, its Neh2 domain may serve as a putative dominant *trans*-repressor of the *PERK* gene. Since eIF2α is known as a direct substrate of PERK, it could also be phosphorylated in the ER response to TU [4], before its contribution to the selective protein translation of ATF4 (as illustrated in Figure 1). Herein, qRT-PCR showed that a time-dependent increment in *ATF4* mRNA levels was induced in *Nrf1/2*^+/+^ cells that had been treated for 8 h to 24 h with TU, which was peaked at 24 h (Figure 3E). Such late stages of TU-inducible *ATF4* response to after 16-h treatment were obviously blocked in *Nrf1α*^−/−^ or *caNrf2^ΔN^* cells. Conversely, *Nrf2*^−/−*ΔTA*^ led to a remarkable, accelerated promotion of *ATF4* expression induced by TU, with an early peak at 12 h (Figure 3E). These results indicate that Nrf2 acts as a dominant trans-repressor of the *ATF4* gene in cellular response to TU, and meanwhile, it is inferable that the transcriptional expression of *ATF4* may be attributable to positive regulation by Nrf1α. Furthermore, TU-inducible mRNA expression levels of *Chop* (as a downstream target gene of ATF4) were markedly reduced by *Nrf1α*^−/−^ or *Nrf2*^−/−*ΔTA*^ to considerable lower extents than those obtained from *Nrf1/2*^+/+^ cells (Figure 3F), but it was almost unaffected by *caNrf2^ΔN^*. This indicates that Nrf1α and Nrf2 contribute to the transcriptional regulation of the *Chop* gene.

Close examinations of the two other ER-stress signaling arms ATF6 and IRE1 also showed distinct time-dependent induction of their mRNA expression by TU treatment of *Nrf1/2*^+/+^ cells, respectively with different peaks at 12 h or 20 h (Figure 3G,H). Such significantly TU-induced increases in the *ATF6* mRNA expression, as well as its basal expression, were substantially suppressed by *Nrf1α*^−/−^ and also mostly inhibited by *Nrf2*^−/−*ΔTA*^ (Figure 3G). This indicates that Nrf1α and Nrf2 are required for regulating transcriptional expression of the *ATF6* gene. However, the contribution of Nrf2 to this response is limited to a certain extent, because *caNrf2^ΔN^* caused no changes in both the basal and TU-stimulated expression of *ATF6*, when compared to those of *Nrf1/2*^+/+^ cells (Figure 3G). Furtherly, induction of *IRE1* mRNA expression by TU, but not its basal expression, was significantly inhibited in *Nrf1α*^−/−^ cells (with a hyper-expression of Nrf2), but not recovered by *Nrf2*^−/−*ΔTA*^ (Figure 3H). This implies that both Nrf1α and Nrf2 contribute to the *IRE1* transcriptional expression. As such, it is intriguing to note that the late-stage induction of *IRE1* after 20-h TU treatment was also reduced by *Nrf2*^−/−*ΔTA*^, by comparison with the control values of *Nrf1/2*^+/+^ cells (Figure 3H). Contrarily, *caNrf2^ΔN^* led to rather significant elevations in both basal and TU-inducible *IRE1* mRNA levels, with an early higher peak that was stimulated at 8-h TU treatment and then maintained to 12 h, after being abruptly lowered to similar levels to the late-stage induction of *Nrf1/2*^+/+^ cells (Figure 3H). From these collective data, it is hence inferable that dual opposing roles of Nrf2 in the *IRE1* transcriptional response to TU, as well as its basal responsive expression, may depend on the presence of distinct functional domains within this CNC-bZIP factor and/or their biochemical modifications by putative signaling, albeit the detailed mechanisms are unknown. Besides, the transcriptional expression of *XBP1* mRNA as a direct substrate of IRE1 was also analyzed by qRT-PCR. The results disclosed that an accelerated increase in *XBP1* mRNA expression to a 4-fold maximal value was induced by TU after 8-h treatment of *Nrf1/2*^+/+^ cells (Figure 3I). By comparison with wild-type, TU-inducible *XBP1* expression was significantly decreased by *Nrf1α*^−/−^, and also partially reduced by *Nrf2*^−/−*ΔTA*^ or *caNrf2^ΔN^*. Thereby, it is postulated that Nrf1α and Nrf2 are involved in coregulating the responsive expression of the *XBP1* gene to TU.

Next, examinations of ARE-driven genes by qRT-PCR revealed that TU treatment caused distinct time-dependent induction of *GCLM* and *HO-1* expression in *Nrf1/2*^+/+^ cells (Figure 3J,K). By contrast, induction of *GCLM* and *HO-1* by TU, as well as their basal mRNA expression, was further significantly incremented in *Nrf1α*^−/−^ cells (retaining hyper-expression of Nrf2). Furtherly, *caNrf2^ΔN^* also gave rise to substantial increases in basal and TU-induced mRNA levels of *GCLM*, rather than *HO-1* (Figure 3J,K). Conversely, *Nrf2*^−/−*ΔTA*^ led to obvious decreases in both basal and TU-induced mRNA levels of *HO-1*, but not of *GCLM* (Figure 3J,K). These imply that transcriptional responses of *HO-1* and *GCLM* to TU are mediated dominantly by Nrf2, albeit Nrf1 partially contributes to this antioxidant response. In addition, the TU-inducible expression of *HO-1* is also partially reduced by *caNrf2^ΔN^* (Figure 3K), implicating a possible attribution to the loss of the Neh2 domain from Nrf2. Together, these demonstrate that the transcriptional expression of *Nrf1*, *Nrf2*, and their co-target genes *GCLM* and *HO-1*, is differentially induced by an ER stressor, as accompanied by distinct activation of integral UPR signaling networks. 

### 3.4. Distinct Contributions of Nrf1α and Nrf2 to the Protein Expression of Antioxidant Responsive Genes to TU that Serves as a Classic ER Stressor

**Figure 4:**
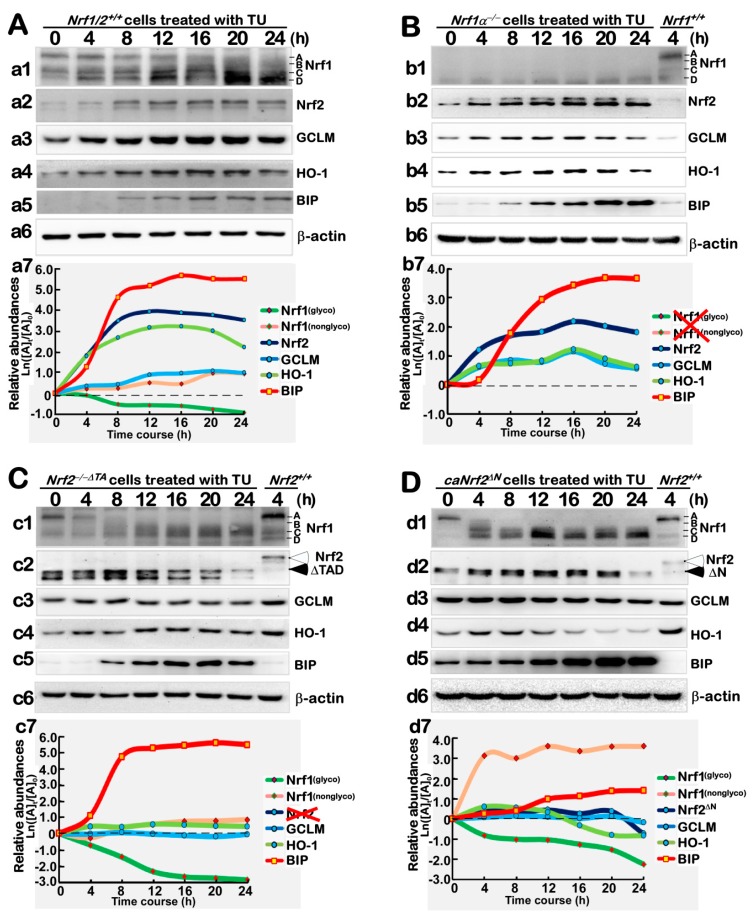
TU-inducible changes in the protein expression of antioxidant genes. Different cell lines of *Nrf1/2*^+/+^ (**A**), *Nrf1α*^−/−^ (**B**), *Nrf2*^−/−*ΔTA*^ (**C**) and *caNrf2^ΔN^* (**D**) were or were not treated with 2 μg/mL TU for indicated lengths of time from 0 to 24 h. The TU-inducible changes in the protein expression of distinct responsive genes were then determined by Western blotting with the indicated antibodies against Nrf1, Nrf2, GCLM, HO-1, BIP/GRP78 or β-actin. The intensity of immunoblots representing different protein expression levels was also quantified by the Quantity One 4.5.2 software, and then shown graphically herein. Note: two big red crosses represent losses of Nrf1 and Nrf2 (*b7* and *c7*), respectively. In addition, no obvious differences between untreated (i.e., NC) and vehicle (i.e., 0.1% DMSO)-treated protein levels of BIP, HO-1, Nrf1 and Nrf2 in wild-type HepG2 cells were visualized by Western blotting (as shown in supplemental Figure S2).

Here, we further determined changes in the time-dependent expression of Nrf1 and Nrf2 proteins, alongside their downstream antioxidant genes *GCLM* and *HO-1*, in different genotypic cellular responses to ER stress induced by TU for distinct lengths of time from 4 h to 24 h. Western blotting of *Nrf1/2*^+/+^ cells, that had been treated by TU (as a specific inhibitor of oligosaccharyl transferases to block the N-glycosylation of newly-synthesized polypeptides that occurs specifically in the ER lumen), revealed that abundance of the full-length Nrf1 glycoprotein-A was gradually decreased from 8 h to its disappearance (Figure 4A, *a1*). Instead, the abundances of deglycosylated and processed Nrf1 protein-C/D, including Nrf1^ΔN^, were relatively incremented as the TU treatment time was increased. By sharp contrast, all those Nrf1α-derived longer isoforms were completely abolished by a specific knockout of *Nrf1α*^−/−^ (Figure 4B, *b1*). Such Nrf1α-derived protein patterns were modestly influenced by *Nrf2*^−/−*ΔTA*^ or *caNrf2^ΔN^*, because both mutants gave rise to an obviously- accelerated disappearance of Nrf1 glycoprotein-A from within 4 h to 8 h of TU treatment (Figure 4C, *c1*; Figure 4D, *d1*), when compared with its extant presence in *Nrf1/2*^+/+^ cells (Figure 4A, *a1*). Conversely, this disappearance of Nrf1 glycoprotein-A was, rather, replaced by additionally accelerated abundances of Nrf1 protein-C/D isoforms in *caNrf2^ΔN^* cells (Figure 4D, *d1*). Together, these demonstrate that the glycosylation, deglycosylation and proteolytic processing of Nrf1 (within and around the ER) are tightly regulated by the TU-induced stress response signaling. Of note, abundances of Nrf1 and its isoforms are also determined by both the translational and transcriptional regulation of Nrf1 in the ER response to TU, part of which is also mediated by Nrf2.

Indeed, it is true that a gradual increment in Nrf2 protein abundances resulted from 4 h to 24 h TU-treatment of *Nrf1/2*^+/+^ cells (Figure 4A, *a2*). More intriguingly, a major processed isoform of Nrf2 was gradually incremented with the increasing time of the TU treatment of *Nrf1α*^−/−^ cells (Figure 4B, *b2*). Similar observations were also represented in *caNrf2^ΔN^*, but not *Nrf2*^−/−*ΔTA*^, cells (*cf*. Figure 4D, *d2* with Figure 4C, *c2*). Hence, it is inferable that the putative proteolytic processing of Nrf2 may occur through and within its N-terminal Neh2 domain, and this process appears to be regulated by Nrf1α, albeit the detailed mechanism requires to be elucidated. 

Further examinations of antioxidant protein expression unraveled that distinct time-dependent increments of GCLM and HO-1 were significantly induced by TU treatment in *Nrf1/2*^+/+^ cells (Figure 4A, *a3*, *a4*). Similar induction of GCLM and HO-1 by TU was also observed in *Nrf1α*^−/−^ cells (Figure 4B, *b3*,*b4*), but was obviously reduced by *Nrf2*^−/−*ΔTA*^ (Figure 4C, *c3*,*c4*,*c7*), compared with the controls from *Nrf1/2*^+/+^ cells. However, *caNrf2^ΔN^* cells displayed almost no changes in GCLM protein (Figure 4D, *d3*), but this was accompanied by a modest decrease in HO-1 protein expression (Figure 4D, *d4*) as consistent with its mRNA expression levels (Figure 3K). Collectively, these results demonstrate that, although Nrf2 is negatively regulated by Nrf1, the former Nrf2 makes a major contribution to regulating the expression of *GCLM* and *HO-1* genes, possibly through its N-terminal Neh2 domain. 

Western blotting of the ER responsive chaperone BIP showed that its protein abundance was increased in a time-dependent manner from 4 h to 24 h of TU treatment of *Nrf1/2*^+/+^ cells (Figure 4A, *a5*). Interestingly, remarkable increments in BIP protein levels were also presented in TU-treated *Nrf1α*^−/−^ and *Nrf2*^−/−*ΔTA*^ cells (Figure 4B, *b5*; Figure 4C, *c5*), albeit its mRNA expression levels were lowered to different extents in these two deficient cell lines (Figure 3C). In addition, basal and TU-stimulated abundances of BIP were further strikingly incremented in *caNrf2^ΔN^* cells (Figure 4D, *d5*). Overall, these indicate that Nrf1α and Nrf2 are not essential for mediating this BIP protein expression, albeit both CNC-bZIP factors are involved in this chaperone transcriptional response to TU (as illustrated in Figure 3C), besides the antioxidant response to this ER stressor.

### 3.5. Distinctive Requirements of Nrf1α and Nrf2 in Differential Expression of the ER Stress-Responsive Genes Induced by TU

**Figure 5:**
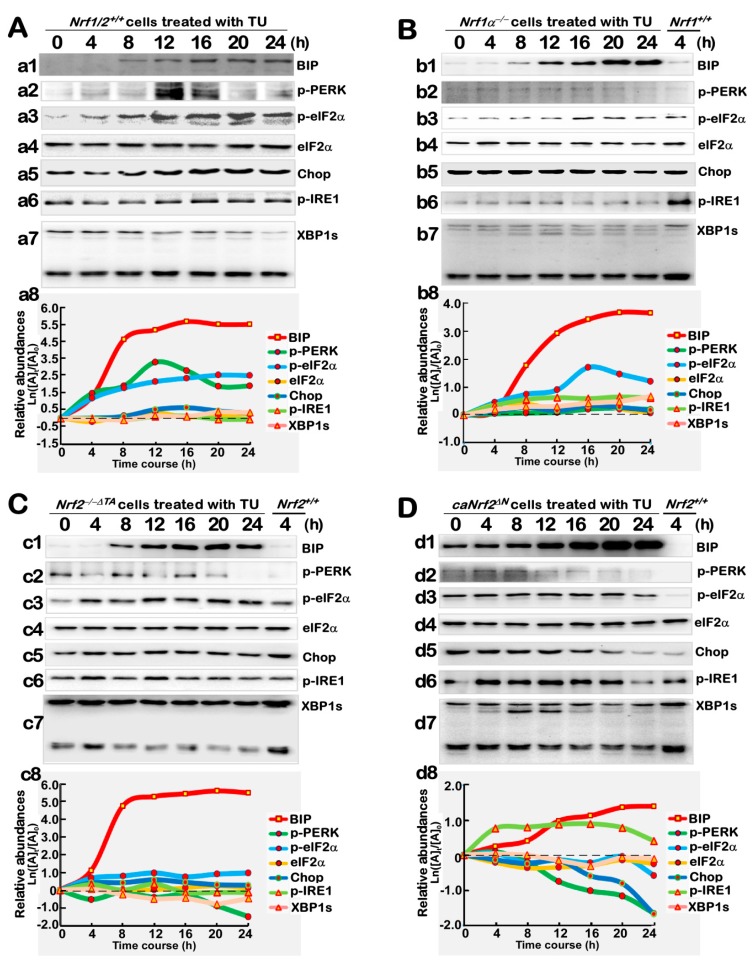
TU-inducible changes in the protein expression of ER stress-responsive genes. Distinct cell lines of *Nrf1/2*^+/+^ (**A**), *Nrf1α*^−/−^ (**B**), *Nrf2*^−/−*ΔTA*^ (**C**) and *caNrf2^ΔN^* (**D**) were or were not treated with TU at 2 μg/mL for the indicated times from 0 to 24 h. The TU-inducible changes in the protein expression of differential responsive genes were determined by Western blotting with the indicated antibodies against BIP (as a positive reference, that was also duplicated from Figure 4), p-PERK, p-eIF2α, eIF2α, Chop, p-IRE1 or XBP1. The intensity of blots representing different protein expression levels was then quantified by the Quantity One 4.5.2 software (Bio-rad, Hercules, CA, USA) and shown graphically. In addition, no obvious differences between untreated and DMSO vehicle-treated BIP abundances in *Nrf1/2*^+/+^ cells were also shown (in supplementary Figure S2).

Clearly, it is known that the chaperone BIP protein is a key sensor to ER stress induced by TU, and expression of its cognate genes as a direct effector is also activated in UPR [1,2,3,4]. Such an ER stress model had been successfully constructed as described above (Figure 3C and Figure 4). Herein, we further examined whether Nrf1α and Nrf2 are required for crucial proteins expression of the UPR signaling cascades. As anticipated, the phosphorylated protein levels of PERK were significantly increased following 12 h to 16 h of the TU treatment of *Nrf1/2*^+/+^ cells (Figure 5A, *a2*). The induction of p-PERK by TU appeared to be blocked in *Nrf1α*^−/−^ and *Nrf2*^−/−*ΔTA*^ cell lines (Figure 5B, *b2*; Figure 5C, *c2*). By contrast, *caNrf2^ΔN^* led to early modest induction of p-PERK by TU at 4 h to 8 h following treatment (Figure 5D, *d2*). These suggest that both Nrf1α and Nrf2 may be required for regulation of the PERK signaling response to TU.

Meanwhile, the total protein abundances of eIF2α (as a main substrate of p-PERK to yield p-eIF2α) were almost unaltered by TU in all the above-described four cell lines (Figure 5A, *a4*; Figure 5B, *b4*; Figure 5C, *c4*; Figure 5D, *d4*). In addition to basal eIF2α auto-phosphorylation, its TU-inducible phosphorylation was also enhanced, as the time of treatment was increased from 4 h to 16 h, and then maintained until 24 h after treatment of *Nrf1/2*^+/+^ cells (Figure 5A, *a3*). However, TU-induced phosphorylation of eIF2α was almost unaffected in *Nrf1α*^−/−^ cells (Figure 5B, *b3*), when compared with the control of 4 h TU-treated *Nrf1/2*^+/+^ cells. Conversely, induction of eIF2α phosphorylation by TU was also partially recovered by inactivation of Nrf2 in *Nrf2*^−/−*ΔTA*^ cells (Figure 5C, *c3*). These results indicate that Nrf1α and Nrf2 may contribute to positive and negative regulation of eIF2α induction by TU, respectively. Yet, *caNrf2^ΔN^* gave rise to an increase in basal eIF2α auto-phosphorylation and its TU-inducible phosphorylation by 20 h of treatment (Figure 5D, d3), by comparison with the control of 4-h TU- treated *Nrf1/2*^+/+^ cells. This intriguing data implies that eIF2α may be negatively regulated by the N-terminal Neh2 domain of Nrf2, besides its transactivation domain (because both domains lacked in *caNrf2^ΔN^* and *Nrf2*^−/−*ΔTA*^ cells, respectively).

Western blotting examination of Chop (as a direct effector of the PERK-eIF2α-ATF4 signaling pathway) revealed that a modest increment in Chop protein levels resulted from 8 h to 24 h of TU treatment of *Nrf1/2*^+/+^ cells (Figure 5A, *a5*). By contrast with wild-type controls, almost no changes in Chop abundances were determined in *Nrf1α*^−/−^ and *Nrf2*^−/−*ΔTA*^ cells, albeit both lines had been treated with TU (Figure 5B, *b5*; Figure 5C, *c5*). However, basal abundance of Chop was obviously elevated by *caNrf2^ΔN^* and almost unaffected by TU within 12 h after this treatment, but thereafter decreased gradually to a similar level to the control value of 4-h TU-treated *Nrf1/2*^+/+^ cells (Figure 5D, *d5*). These indicate that Chop is co-regulated by Nrf1α and Nrf2, but the latter Nrf2 may contribute to a positive regulation of Chop possibly by its Neh2 domain, in the ER-to-nuclear response to the long-term TU-induced stress. In addition, some contradictory data (Figure 3 and Figure 5) were also argued to imply that Nrf1α and Nrf2 might exert combinational, differential and even opposite roles in regulating the PERK-eIF2α- and ATF4/Chop-signaling pathways at distinct layers, e.g., of transcriptional and translational expression, as well as the post-biosynthetic processing of their mRNAs and proteins, within interaction networks.

Next, time-dependent expression of the IRE1/ATF6-XBP1 signaling molecules was examined by Western blotting. The results unraveled that, since the existing auto-phosphorylation of IRE1 has been at a considerably higher level, thus TU only induced a modest increase in its phosphorylated abundance following 12-h treatment of *Nrf1/2*^+/+^ cells (Figure 5A, *a6*). Such lower induction of IRE1 by TU appeared to be almost abolished in *Nrf1α*^−/−^ cells (Figure 5B, *b6*, when compared to the control of 4-h TU-treated *Nrf1/2*^+/+^ cells), but was only slightly recovered by *Nrf2*^−/−*ΔTA*^ from 4 h to 12 h of TU treatment (Figure 5C, *c6*). But, *caNrf2^ΔN^* cells raised a rather higher induction of phosphorylated IRE1 by TU (Figure 5D, *d6*). Together, these indicate that Nrf1α and Nrf2 are involved in regulating the IRE1 signaling response to TU. In addition, it is important to note that the intact XBP1u mRNA, though as a direct substrate of IRE1 to yield an alternatively-spliced XBP1s (besides its mRNA decay [1,2,3,4]), is also transcriptionally regulated by the ATF6 signaling in the ER stress response to TU (as a proposed model in Figure 1). Here, a few of processed XBP1s bands were observed by the Western blotting of *Nrf1/2*^+/+^ cells (Figure 5A, *a7*), and obviously enhanced by TU in either *Nrf1α*^−/−^ or *caNrf2^ΔN^* cell lines (Figure 5B, *b7*; Figure 5D, *d7*), but not determined in *Nrf2*^−/−*ΔTA*^ cells (Figure 5C, *c7*). Conversely, the putative XBPu protein was, rather, reduced by *Nrf2*^−/−*ΔTA*^ only. These results implicate that Nrf2 may make a contribution to regulating the XBP response to TU, albeit the detailed mechanism needs to be further elucidated. 

### 3.6. Almost No Induction of the Proteasomal (PSM) Subunit Genes Regulated by Nrf1 in the Response to TU

**Figure 6:**
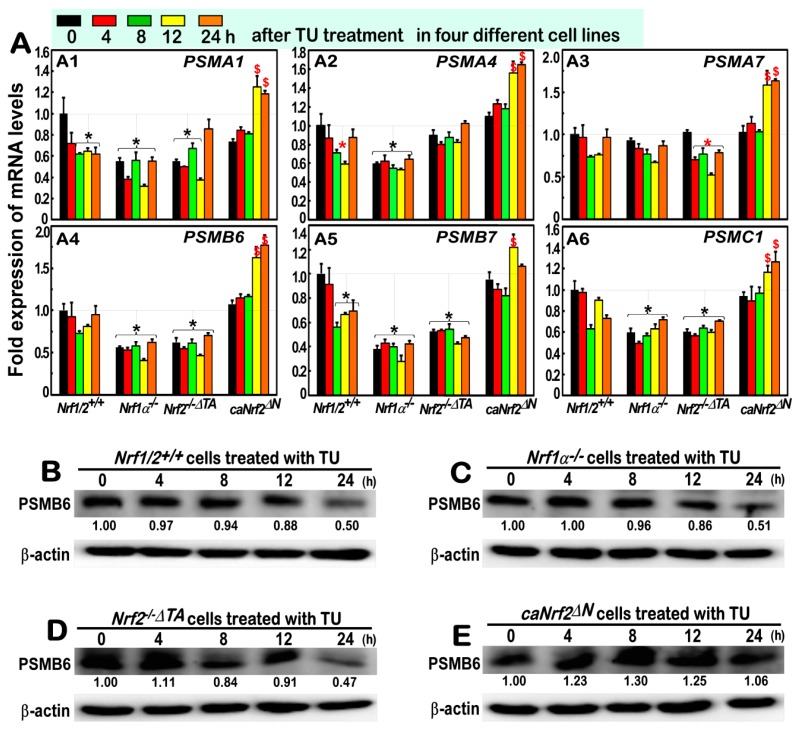
No obvious induction of some examined proteasomal genes by TU. Different cell lines of *Nrf1/2*^+/+^, *Nrf1α*^−/−^, *Nrf2*^−/−*ΔTA*^ and *caNrf2^ΔN^* were or were not treated with 2 μg/mL of TU for the indicated times from 0 to 24 h. The TU-inducible mRNA expression levels of some proteasomal genes, including *PSMA1*, *PSMA4*, *PSMA7*, *PSMB6*, *PSMB7* and *PSMC1*, were analyzed by real-time qPCR (**A**), and Western blotting with indicated antibodies against PSMB6 or β-actin in different cell lines of *Nrf1/2*^+/+^ (**B**), *Nrf1α*^−/−^ (**C**), *Nrf2*^−/−*ΔTA*^ (**D**) and *caNrf2^ΔN^* (**E**). The intensity of blots representing PSMB6 was quantified by the Quantity One 4.5.2 software, and is shown on the bottom. Significant statistical increases were indicated with $, *p* < 0.01, whereas significant decreases were represented by * *p* < 0.01, respectively, relative to the control values obtained from *Nrf1/2^+/+^* cells. In addition, almost no differences in between untreated (i.e., NC) and 0.1% DMSO vehicle-treated mRNA and protein levels of PSMB6 in *Nrf1/2*^+/+^ cells were also determined by qRT-PCR and Western blotting, respectively (as shown in supplemental Figure S2).

As described by us and other groups [22,24,29], it is rather clear that Nrf1, but not Nrf2, exerts an important biological role in the transcriptional expression of all *PSM* genes. Such transcriptional regulation of these *PSM* genes by Nrf1 is accompanied by the induction of all three classic ER-driven stress response signaling pathways mediated by PERK1, IRE1 and ATF6, with the proteasomal compensatory response to limited extents of the proteasomal inhibitors [24]. Herein, we examined whether TU-stimulated Nrf1 and Nrf2 are required for the ER-to-nuclear signaling responses to transactivate *PSM* genes. The results revealed that none of all six *PSM* mRNA expression levels were induced by TU in *Nrf1/2*^+/+^ cells (Figure 6A). Amongst them, both *PSMA1* and *PSMB7* mRNA levels were, however, obviously down-expressed in TU-treated *Nrf1/2*^+/+^ cells (Figure 6A, *a1*, *a5*). 

Further qRT-PCR analysis of *Nrf1α*^−/−^ cells unraveled that the basal mRNA expression levels of *PSMA1*, *PSMA4*, *PSMB6*, *PSMB7* and *PSMC1*, but not *PSMA7*, were significantly down-regulated, although they were unaffected by TU stimulation (Figure 6A). Similar down-regulation of *PSMA1*, *PSMB6*, *PSMB7*, *PSMC1*, but not *PSMA4* or *PSMA7*, was detected in *Nrf2*^−/−*ΔTA*^ cells. However, all these six *PSM* genes were only induced by the long-term exposures of TU in *caNrf2^ΔN^* cells for 12 h to 24 h. This may imply a hitherto unknown mechanism by which the transcriptional expression of 26S proteasomal subunits is also controlled by the constitutive activation of *caNrf2^ΔN^*.

Such being the case, Western blotting analysis of *PSMB6* disclosed that its protein abundance was decreased by TU, with the increasing treatment time of *Nrf1/2*^+/+^ cells (Figure 6B). Similarly, such TU-triggered decreases in *PSMB6* were almost unaltered by either *Nrf1α*^−/−^ or *Nrf2*^−/−*ΔTA*^ cells (Figure 6C,D), but markedly reversed by *caNrf2^ΔN^* cells with a modest increase (Figure 6E). Collectively, distinct contributions of Nrf1α and Nrf2 to basal, rather than TU-inducible, expression of some *PSM* genes are demonstrated. However, Nrf2 might also exert opposing roles in this event, depending on its functional domains within different responsive contexts.

### 3.7. Nrf1α Is More Potent Than Nrf2 at Mediating Cytoprotective Response against the Cytototic Effects of TU

**Figure 7:**
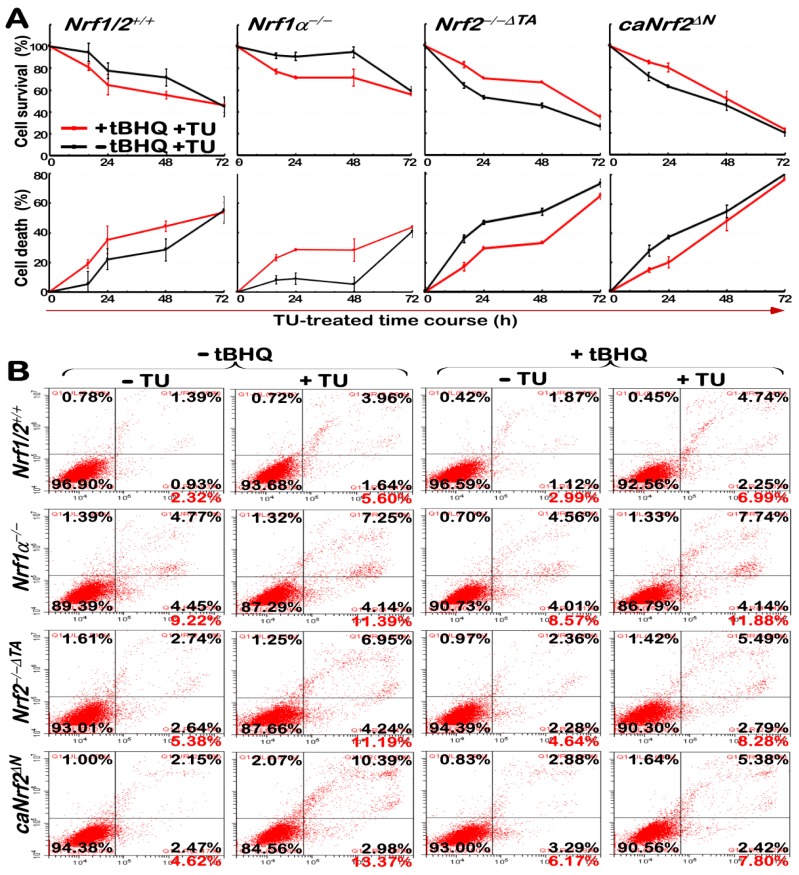
Nrf1 is more potent than Nrf2 at mediating the putative cytoprotective response to TU. (**A**) Four different cell lines *Nrf1/2*^+/+^, *Nrf1α*^−/−^, *Nrf2*^−/−*ΔTA*^ and *caNrf2^ΔN^* were or were not pretreated with 50 μmol/L tBHQ or 0.1% DMSO vehicle (i.e., −tBHQ) for 16 h, before they were or were not treated with 2 μg/mL TU for the indicated times from 0 to 72 h. These cell survivals and deaths were then evaluated by using the MTT-based cell proliferation and cytotoxicity assays. (**B**) After pretreatment of the above four cell lines with +tBHQ (50 μmol/L) or without −tBHQ (0.1% DMSO) for 16 h, they were or were not treated with 2 μg/mL of TU for an additional 48 h. Subsequently, these cells were incubated in a binding buffer containing both Annexin V-FITC and propidium iodide (PI) for 15 min, before being subjected to flow cytometry analysis of apoptosis. The resulting data were analyzed by the FlowJo 7.6.1 software (also see Supplemental Figure S3).

Most studies by using mouse embryonic fibroblasts (MEFs) have revealed that Nrf2 mediates an adaptive response to sulforaphane that protects fibroblasts in vitro against the cytotoxic effects of electrophiles, peroxides, redox-cycling agents and other reagents [39,40]. Like Nrf2, Nrf1 is also activated in a similar response to tBHQ [20]. Herein, we determined whether either Nrf1 or Nrf2 mediates a similar adaptive response to tBHQ, and thus protects human hepatocellular carcinoma cell lines (with the presence or absence of both CNC-bZIP factors) against the cytototic effects of TU. Interestingly, as shown in Figure 7A, pretreatment of tBHQ for 16 h obviously promoted the TU-induced death of either *Nrf1/2*^+/+^ or *Nrf1α*^−/−^ cell lines (albeit Nrf2 is preserved or hyper-activated in both lines, respectively). Conversely, the survival of both *Nrf2*^−/−*ΔTA*^ and *caNrf2^ΔN^* cell lines (because Nrf1 is also retained or incremented, respectively) was thereby enhanced by tBHQ pretreatment in a putative cytoprotective response against the cytototic effects of TU. These results reveal that Nrf1 is more potent than Nrf2 at mediating the cytoprotective response to TU.

Further experimental evidence obtained from the flow cytometry analysis of apoptotic cells (Figure 7B and Figure S3), unraveled that only a small number of apoptotic cells indeed resulted from the cytototic effect of TU on *Nrf1/2*^+/+^ cells. Such the TU-induced apoptosis of *Nrf1/2*^+/+^ cells was also marginally enhanced by pretreatment with tBHQ. By contrast, TU stimulated a relative stronger apoptotic effect on *caNrf2^ΔN^* cells (in which Nrf1 and Nrf2 were up-expressed or activated), but such TU-induced apoptotic effect of caNrf2^ΔN^ cells was significantly suppressed by tBHQ pretreatment (Figure 7B and Figure S3). Similarly, TU also enabled the triggering of stronger cytototic effects on either *Nrf1α*^−/−^ and *Nrf2*^−/−*ΔTA*^ cell lines, leading to almost equivalent extents of apoptosis. However, it is intriguing that the TU-triggered apoptosis of *Nrf2*^−/−*ΔTA*^ cells, but not of *Nrf1α*^−/−^ cells, was strikingly repressed by tBHQ pretreament (Figure 7B and Figure S3). Notably, almost no changes in apoptosis resulting from only the 16-h tBHQ pretreament of all of the above four cell lines were observed by comparison with their respective auto-apoptosis. Together, these results demonstrate that Nrf1 is more potent than Nrf2 at mediating the cytoprotective response against the cytototic effects of TU.

### 3.8. The Intracellular ROS Levels Are Increased in Nrf1α^−/−^ cells, but Decreased in Nrf2^−/−ΔTA^ Cells, with Different Responses to TU Alone or Plus tBHQ

**Figure 8:**
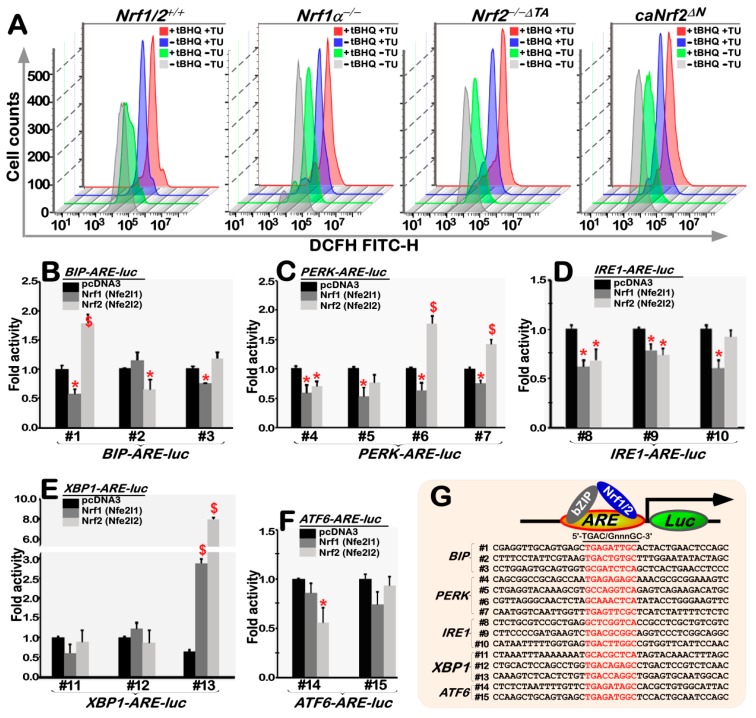
Requirement of Nrf1 and Nrf2 for mediating antioxidant cytoprotective responses to stress stimulated by TU alone or plus tBHQ. (**A**) After reaching 70% of their confluence, experimental cells with distinct genotypes of *Nrf1/2*^+/+^, *Nrf1α*^−/−^, *Nrf2*^−/−*ΔTA*^ or *caNrf2^ΔN^* were allowed for growth in fresh media with tBHQ (50 μmol/L) or the vehicle DMSO (i.e., −tBHQ) for 16 h, before being treated with 2 μg/mL of TU or without this chemical (i.e., −TU) for an additional 48 h. Thereafter, the cells were subjected to a flow cytometry analysis of the intracellular ROS-DCFH-DA fluorescent intensity. The resulting data were further analyzed by the FlowJo 7.6.1 software. (**B**–**F**) *Nrf1/2*^+/+^ HepaG2 cells were co-transfected with each of those indicated *ARE-Luc* or non-ARE-*Luc* (as a background control) plasmids, together with an expression constructed for Nrf1, Nrf2 or an empty pcDNA3.1 vector. The luciferase activity was normalized to their internal controls and corresponding backgrounds obtained from the co-transfection of cells with non-ARE reporter and each of the expression constructs. The results were calculated as a fold change (mean ± SD, n = 6) relative to the basal activity (at a given value of 1.0) obtained from the transfection of cells with the empty pcDNA3.1 vector and each of the ARE-driven luciferase plasmids. All the data shown herein are representative of at least three independent experiments undertaken on separate occasions that were each performed in triplicate. Significant differences in the ARE-driven transactivity mediated by Nrf1 and/or Nrf2 were subjected to statistical analysis; there are significant increases ($ *p* < 0.05) and decreases (* *p* < 0.05), by comparison to their basal levels. (**G**) 15 of the indicated ARE-adjoining sequences from UPR-related gene promoters were inserted in the pGL3-Promoter vector, which served as *ARE-Luc* reporters.

Under almost untreated, normal conditions, *Nrf1α*^−/−^ cells were maintained at higher ROS levels, whilst *Nrf2*^−/−*ΔTA*^ cells were rather preserved at relatively lower ROS levels, when compared to those examined in the wild-type *Nrf1/2*^+/+^ cells (Figure 8A, Figures S4 and S5); this is fully consistent with our previous observation [34]. However, it is, to our surprise, found that *caNrf2^ΔN^* cells were also saved under higher ROS conditions similar to those measured from *Nrf1α*^−/−^ cells. Amongst all four examined cell lines, only *Nrf1/2*^+/+^ cells, rather than *Nrf1α*^−/−^, *Nrf2*^−/−*ΔTA*^ or *caNrf2^ΔN^* cells, gave rise to an obvious reduction of these ROS levels (with their images left-shifted) by the exposure to tBHQ (Figure 8A, Figures S4 and S5). This implies an effective cooperation between Nrf1 and Nrf2 in mediating antioxidant responses to this redox inducer. 

Under non-tBHQ-pretreated conditions, the intracellular ROS levels were marginally reduced by the TU treatment of *Nrf1/2*^+/+^ and *Nrf1α*^−/−^, but rather modestly increased in TU-treated *Nrf2*^−/−*ΔTA*^ or *caNrf2^ΔN^* cells (Figure 8A, Figures S4 and S5). Under tBHQ-pretreated conditions, TU treatment of *Nrf1α*^−/−^ and *caNrf2^ΔN^* cells caused an obvious decrease in their ROS levels, whilst a relative increase in the other ROS levels yielded in *Nrf1/2*^+/+^ and *Nrf2*^−/−*ΔTA*^ cells. Collectively, these results imply that distinct cellular redox responses are triggered by TU stimulation of different mechanisms involving Nrf1 and Nrf2, that exert overlapping or opposing effects on the transcriptional profiling. 

### 3.9. Nrf1 and Nrf2 Bi-Directionally Mediate Transcriptional Expression of Distinct ARE-Driven UPR-Luc Reporter Genes

To determine whether Nrf1 and/or Nrf2 directly mediate the transcriptional expression of some key genes required for UPR, thereby we established 15 of the indicated *ARE-Luc* reporter genes (Figure 8B–G), in which the consensus ARE-adjoining sequences from the promoter regions of *BIP*, *PERK*, *IRE1*, *ATF6* and *XBP1* were inserted. Subsequently, co-transfection experiments revealed that both Nrf1 and Nrf2 significantly transactivate the expression of *XBP1-ARE* (#13)*-Luc* (Figure 8E). All the other reporter genes, such as *BIP-ARE* (#1,#3)*-Luc*, *PERK-ARE* (#4–7)*-Luc*, *IRE1-ARE* (#8–10)*-Luc* and *ATF6-ARE* (#14)*-Luc* were down-regulated by Nrf1 (Figure 8B–F). By contrast, Nrf2 enabled bidirectional regulation of *BIP-ARE* (#1,#2)*-Luc*, *PERK-ARE* (#4,#6,#7)*-Luc* and *IRE1-ARE* (#8,#9)*-Luc* transcriptional expression (Figure 8B–D). These results demonstrate that Nrf1 and Nrf2 have more potent abilities to contribute to the bi-directional regulation of these critical UPR genes. Of note, it cannot be ruled out that the expression of these *ARE-Luc* reporter genes may be also determined by other factors in different contexts.

## 4. Discussion 

**Figure 9:**
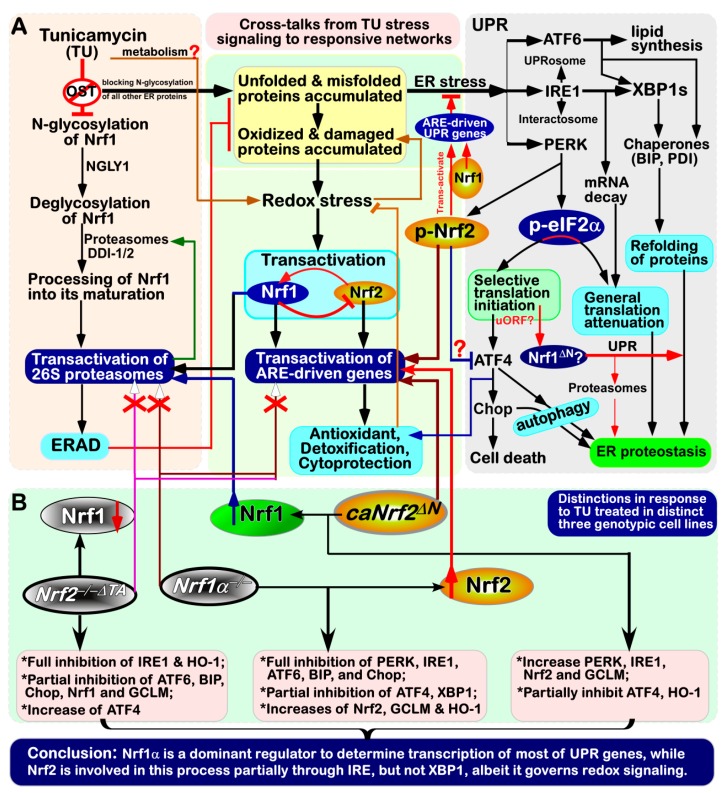
Cross-talks between distinct signaling pathways activated within different ER-to-nuclear responsive networks to the classic ER stressor TU. (**A**) There exist multiple cross-talks from the TU-induced stress signaling to different ER-to-nuclear responses. Inhibition of Nrf1 N-glycosylation by TU leads to subsequent blockage of its deglycosylation and even proteolytic processing to yield a mature CNC-bZIP factor, before transactivating transcriptional expression of the proteasomal (*PSM*) genes. On the contrary, disruption of Nrf1-mediated proteasomal degradation (e.g., ERAD) leads to aberrant accumulation of oxidized, damaged and misfolded proteins, in addition to other TU-led, non-glycosylated and unfolded proteins. These, together, result in classic ER stress along with redox stress. Of note, distinct and even opposing roles of Nrf1 and Nrf2, as well as both inter-regulatory effects on cytoprotection against ER redox stress, are integrally unified in different cellular signaling responses within distinct gene-regulatory networks. (**B**) Our evidence demonstrates that Nrf1 acts as a dominant regulator of most of UPR-target genes, apart from its negative regulation of Nrf2. By contrast, Nrf2, besides being a direct substrate of PERK, is also involved in this response to TU, partially by the IRE1 signaling pathway, but not by its downstream XBP1. Although Nrf2 governs the transcriptional expression of Nrf1 and some co-target antioxidant genes, it also contributes to the negative regulation of ATF4, that is selectively translated by the eIF2α-based initiation machinery. Furthermore, Nrf1 is similar to ATF4, in that it is here predicted that both contain an upstream open reading frame (uORF) within their full-length mRNA transcripts. Thereby, it is inferable that the TU-induced ER stress might confer it to be translated into an N-terminally-truncated Nrf1 protein (i.e., Nrf1^ΔN^) that lacks the ER-targeting peptide and its adjacent N-terminal region, so that it freely translocates the nucleus to regulate cognate target genes. Notably, activated or blocked nodes in the signalling cascades are denoted by arrows (→) or bars (⊥), respectively. Some up-regulated (↑) or down-regulated (↓) nodes are also indicated, whereas complete abolishment is represented by red crosses (✕). In addition, the open questions (?) arising from this study remain to be solved.

In the present study, it is convincingly demonstrated that there exists a bi-directional crosstalk between UPR-triggered signaling and ARE-driven cytoprotective responses to the ER stressor TU (Figure 9). Importantly, we have also presented the evidence that opposite roles of Nrf1 and Nrf2 are unified to coordinate distinct cellular responses to TU, leading to a differential activation of ER- driven stress signaling networks. Of note, loss of Nrf1 down-regulates expression of antioxidant, detoxification and 26S proteasomal genes, resulting in severe oxidative stress and concurrently ER stress [27,32]. The latter pathophysiological event is primarily attributable to the disruption of protein folding within the ER and the dysfunction of ERAD, such that unfolded and misfolded proteins, along with oxidized and damaged proteins, are aberrantly accumulated within the ER, so as to become a proteotoxic stress on cells. Consequently, the canonical UPR signaling pathways are activated by three ER-tethered transducers PERK, IRE1 and ATF6 (Figure 1; Figure 9), in order for the ER adaptive remodeling to diminish loading of those nascent polypeptides, remove aberrantly misfolded proteins, and then restore itself an intact biological function of this organelle [41,42]. This notion is supported by the evidence showing that endogenous ER stress signaling to activate UPR occurred in the steatotic hepatocytes with a homozygous knockout of *Nrf1*^−/−^ [27], but not of *Nrf2*^−/−^ [12]. A similar ER stress-inducible response was also further enhanced by proteasomal inhibition of the heterozygous *Nrf1*^+/^*^−^* livers, when compared with wild-type [27]. Thereby, Nrf1 plays an essential role in maintaining the homeostasis of ER in cells, but also its functional loss in mice results in the ER transformation and proliferation of *Nrf1*^−/−^ cells in conditional knockout mice, that are spontaneously developed with non-alcoholic steatohepatitis and liver cancer [32]. Such phenotypes are embodied as a pathological consequence of chronic ER stress overstimulation and prolonged UPR signaling activation, which occurred concomitantly with severe oxidative stress, which altogether ultimately leads to carcinogenesis [43,44,45].

Nrf1 is a *bona fide* moving transmembrane protein with dynamic membrane topologies (that are somewhat similar to, but different from, those of PERK, IRE1 and ATF6 within and around the ER, as deciphered in Figure 1). Notably, distinct topovectorial processes of Nrf1 (and/or its derivate isoforms) determine its post-synthetic modification and its transactivity to mediate distinct target genes (e.g., *PSM*) [24,37]. Interestingly, our evidence has been provided showing that the canonical UPR signaling by PERK, IRE1 and ATF6 to the differential expression of distinct responsive genes is activated by the TU treatment of different cell lines, with the presence or absence of Nrf1 and Nrf2. The classic TU-induced ER stress-responsive signaling is accompanied by the transcriptional expression of *Nrf1* and *Nrf2*, as well as both co-target genes *GCLM* and *HO-1* (in this study). This appears to raise a paradoxical question about Nrf1, because N-glycosylation of this CNC-bZIP protein (that is newly synthesized in the ER lumen) is sufficiently blocked by TU, with secondary inhibition of its ensuing deglycosylation and its proteolytic processing to yield an N-terminally- truncated mature factor in close proximity to membranes. This inhibitory effect of TU on Nrf1 is also further supported by no induction of all those examined *PSM* genes regulated by Nrf1, rather than Nrf2, leading to ERAD dysfunction so as to exacerbate the accumulation of those misfolded, oxidized and damaged proteins by the stressor TU. However, in a feedback regulatory response to mitigate this deteriorating ER proteotoxic stress, Nrf1 is allowed for transcriptional activation by Nrf2, albeit the latter Nrf2 is negatively regulated by the former Nrf1 ([34], and this study). This is based on the fact that *Nrf1* is identified as a direct target gene of Nrf2 [34], besides itself factor, while Nrf2, as a direct substrate of PERK, is activated by TU [6,11,12]. Hence, it is surmised that the *de novo* biosynthetic Nrf1 protein, as a consequence, is hardly allowed to translocate the deteriorating ER lumen stressed by TU, so that it could be rapidly released from such proteotoxic ER compartments and then subjected to proteolytic processing of nascent Nrf1 polypeptides by cytosolic proteasomes and/or other proteases, in order to yield multiple isoforms with distinct and even opposing trans-activity to regulate the differential expression of a different subset of target genes. Furthermore, translational expression of Nrf1 could also be selectively initiated by a putative mechanism accounting for ATF4, c-Myc and C/EBP [46,47,48] (Figure 9). This is, therefore, predicted to enable for selective translational expression of Nrf1 to switch for its upstream open reading frames (uORFs) to the main open reading frames (mORF), particularly in the intracellular response to ER stress [9]. The resulting selective translation of longer Nrf1 transcripts may be conferred to generate a relatively short N-terminally-truncated Nrf1 protein (e.g., Nrf1^ΔN^), that lacks the ER-targeting peptide, and thus freely translocates the nucleus to regulate cognate target genes. Such being the case, similar uORFs (and/or its products Nrf1u) are also postulated to become a critical player, as a molecular pathophysiological switch driving carcinogenesis or other degenerative diseases [46,47,48]. 

Although the above relevant detailed mechanisms warrant to be further elucidated, we have herein presented the experimental evidence demonstrating that Nrf1 is significantly increased at its transcriptional and translational expression levels in TU-stimulation of *Nrf1/2*^+/+^ cells, particularly of *caNrf2^ΔN^* cells (with an enhanced constitutive expression of Nrf2). This implies that a portion of non-glycosylated Nrf1 protein and its subsequent processed isoforms are incremented in a feedback regulatory circuit. However, it is inferable that a fraction of such immature non-glycosylated and processed proteins of Nrf1 and its derivates (that are generated under the TU-stress conditions) could only retain a weak activity to mediate target genes, relative to another distinctive fraction of its deglycosylated and mature processed proteins (that are generated in TU-untreated cells). In fact, this notion is fully in agreement with the convincing evidence that inhibition of peptide:N-glycosidase (PNG-1/NGLY1, an evolutionarily-conserved deglycosylation enzyme) leads to inactivation of Nrf1, thus a down-regulated expression of target *PSM* genes to potentiate the cytotoxicity of proteasomal inhibitors [49]. Besides, the NGLY1-NRF1 pathway has been revealed to exert an additional novel function in mitochondrial homeostasis and inflammation pathogenesis [50]. Similarly, disruption of deglycosylation of misfolded N-glycosylated proteins to inactivate Nrf1 has been recently unveiled by a loss-of-function of *NGLY1*^−/−^, as a homozygous mutant condition that was characterized by a complex neurological syndrome, hypo- or alacrimia, and also elevated liver transaminases [51,52]. Furtherly, the functional analysis of a *Drosophila* model of NGLY1 deficiency to inactivate its Skn-1 factor also provides insights into therapeutic approaches [53]. The full-length homolog Skn-1A, like Nrf1α, is determined to associate with the ER of *Caenorhabditis elegans* and mediates a cytoplasmic unfolded protein response (UPR^ER^), as well as to promote the longevity [54]. This event occurs to be also regulated by the amino acid sequence editing of Skn-1A by NGLY1 from its glycosylated Asn (-N-S/T-) into acidic Asp (-D-S/T-) residues within this protein, in order to control the transcriptional expression of *PSM* genes in defending against proteotoxic stress [55]. Altogether, these supportive findings are prime to corroborate our pioneering work on Nrf1 glycosylation and deglycosylation to finely tune its transactivation activity [20,56,57].

Notably, the dynamic membrane-topology of Nrf1 to determine its post-synthetic processing and its transactivation activity might be modulated by cholesterol within membranes in the ER-to- nuclear signaling response to cellular stress. This is attributable to the direct binding of Nrf1 to the membrane cholesterol by its five cholesterol-recognized amino acid consensus (CRAC) motifs in this CNC-bZIP protein [37,57,58]. Recently, the ER-associated Nrf1 isalso identified as a vital sensor to cholesterol within membranes [59]. These collective findings demonstrate that Nrf1 plays a central role in maintaining the cholesterol homeostasis by a negative feedback regulatory loop against an additional ER membrane-bound sterol response element binding protein (SREBP2). In reality, the UPR signaling mediated by SREBP (and ATF6) has also been showed to be induced in a lipid stress response [60,61,62]. Activation of SREBP signaling occurs only after being translocated into the Golgi apparatus, in which it is subject to sequential cleavages by Site-1 and Site-2 proteases in a similar fashion to the case of ATF6 (as proposed in Figure 1). However, Nrf1 is neither translocated into the Golgi apparatus and nor cleaved by both Site-1 and Site-2 proteases [38,56]. As a matter of fact, the *bona fide* activation of Nrf1 occurs only after its dynamic flipping out of ER membranes and then by its topology-regulated juxtamembrane proteolytic processing by cytosolic proteasomes and DDI-1/2 proteases to yield a mature CNC-bZIP factor [9,24,37,63]. Besides, SREBP1 was also identified as a direct upstream regulator of Nrf1, which is involved in the mTORC1 signaling response to insulin and the epidermal growth factor [23]. Yet, it is unknown whether there exists a coordinated crosstalk between oxidative stress response and lipid-coupled UPR. As such, it is plausible that Nrf1 senses cholesterol changes in the vicinity of ER membranes. The relevant signals are integrated and then transduced to downregulate the target genes responsible for lipid metabolism. In the meanwhile, the cytoprotective responsive signaling to ER stress enables Nrf1 to be selectively translated by the putative uORF existing within the first exon of its full-length transcripts and/or processed by its topology-regulated juxtamembrane proteolysis. Such a unique mechanism is definitely distinctive from that which accounts for both ATF6 and SREBP1/2 factors in the ER stress responses to unfolded proteins and overloaded lipids. Our transcriptomic sequencing here unraveled that Nrf1α (and Nrf2) may contribute to the basal expression of Site-2 proteases, but it is unknown whether it affects the processing of ATF6 and SREBP1/2. In addition, it should also be noted that Nrf2 is not translocated into the ER lumen; thus it is impossible to be N-glycosylated by the luminal-residing oligosaccharyl transferases (OSTs), albeit it is directly phosphorylated by PERK with the enzymatic central of this kinase that is topologically positioned on the extra-luminal side of ER membranes. Besides, Nrf1 and Nrf2 may be activated by the oxidative stress concomitantly resulting from the proteotoxic stress. 

Herein, it is of a crucial significance to reveal that specific knockout of *Nrf1α*^−/−^ leads to an aberrant hyper-expression of Nrf2 and both cognate targets GCLM and HO-1, but as accompanied by down-expression of most UPR-target genes (in this study). Conversely, inactivation of Nrf2 by *Nrf2*^−/−*ΔTA*^ down-regulates its target genes *GCLM*, *HO-1* and *Nrf1*, with a concurrent inhibition of some UPR-target genes, such as *IRE1*, *ATF6*, *BIP* and *Chop*. However, ATF4 is rather up-regulated strikingly by the inactivation of Nrf2, while their upstream PERK expression is almost unaffected by the loss of Nrf2. These findings, together with our previous work [24,34], demonstrate that Nrf1α does not only dictate the specific functioning of Nrf2 to be elevated in antioxidant responses, and also act as a dominant player in the regulation of most UPR-target genes (that contain the *ARE* consensus sequences in their promoter regions, as listed in Table 2). As anticipated, the indicated ARE-driven reporter assays revealed that Nrf1and/or Nrf2 have the ability to make bi-directional contributions to the positive or negative regulation of the critical UPR genes, such as *BIP*, *PERK*, *XBP1* and *IRE1*. In addition, it should also be noted that the human *ATF6-ARE-Luc* is negatively regulated by Nrf1, but not Nrf2, even though the mouse ATF6 was previously reported as a direct target gene, that is likely to be positively regulated by Nrf1 [64]. The latter authors assumed that down-expression of ATF6 in liver-specific *Nrf1*^−/−^ mice resulted in severe endogenous proteotoxic stress, leading to spontaneous development of NASH and liver cancer [64]. However, such negative regulation of lipids by ATF6 (albeit as a master regulator of lipid biosynthesis) is not yet identified. 

Although our evidence discovers that Nrf2 can serve as a direct upstream regulator to mediate the transcriptional expression of *BIP-ARE-Luc*, *PERK-ARE-Luc* and *XBP1-ARE-Luc* reporters, in fact this CNC-bZIP factor Nrf2 is only partially involved in mediating the expression of some UPR genes by controlling the transcription of *Nrf1* and *IRE1*, but not *XBP1*. However, the expression of ATF4 is down-regulated by the *caNrf2^ΔN^* mutant, albeit with the up-regulation of *PERK* (and *IRE1*). This also implies a putative mechanism accounting for Nrf2 (as a direct substrate of PERK) to compete against the PERK-eIF2α-ATF4 signaling in a feedback circuit (Figure 9). Furthermore, partial inhibition of HO-1 by the *caNrf2^ΔN^* mutant indicates a constitutive loss of its N-terminal Neh2 function as an extra unidentified nuclear transactivation domain after Nrf2 enters the nucleus. 

## 5. Conclusions

The experimental evidence has been here presented demonstrating a unity of distinct and even opposing roles of Nrf1 and Nrf2 in discrete cellular signaling responses to ER stress induced by TU. This results in a differential activation of the ER-to-nuclear signaling networks to regulate different subsets of target genes. There exist multiple cross-talks between UPR- and ARE-driven signaling cascades, aiming to maintain cellular redox, protein and lipid homeostasis. Taken together, these responses enable cell fate decisions to be modulated under ER stress conditions [65], as discovered for its homologous Skn-1 in *Caenorhabditis elegans* [18]. Importantly, Nrf1α (and/or its derivates) is a dominant regulator to determine transcriptional expression of most of UPR-target genes, whereas Nrf2 is also involved in the ER-to nuclear response partially through IRE1, but not its substrate XBP1, albeit it is a central player in governing redox signaling networks. Interestingly, we have discovered that Nrf1 and Nrf2 have a potential to make bi-directional contributions to the positive and/or negative regulation of certain key UPR-target genes, such as *BIP*, *PERK*, *IRE1*, *XBP1* and *ATF6*. Amongst them, the transcriptional expression of ARE-driven *BIP-*, *PERK-* and *XBP1-Luc* reporter genes was mediated directly by either Nrf1 or Nrf2. Of note, although there exists an effective cooperation of between Nrf1 and Nrf2 in regulating their downstream genes, Nrf1α is more potent than Nrf2 at mediating the adaptive cytoprotective responses against the cytotoxic effects of TU alone plus tBHQ. This is also supported by the evidence that the intracellular reactive oxygen species levels are increased in *Nrf1α*^−/−^ cells, but decreased in *Nrf2*^−/−*ΔTA*^ cells. Such being the case, it should also be noted that there exist a very few of the somewhat contradictory data presented herein, that are open to debate. This might also be attributable to the complexity that Nrf1α and Nrf2 could exert combinational, differential and even opposite roles in regulating distinct ER-to-nuclear responsive signaling induced by TU. These complex signaling cascades are further integrated with distinct, gene-regulatory networks, each of which also occurs at multiple layers, such as transcriptional and translational expression, as well as the post-biosynthetic processing of their mRNAs and proteins, within hierarchical interaction networks.

## Figures and Tables

**Table 1:** The primer pairs used for qRT-PCR analysis.

ID	Name	Forward Primers (from 5′ to 3′-ends)	Reverse Primers (from 5′ to 3′-ends)
60	β-actin	CATGTACGTTGCTATCCAGGC	CTCCTTAATGTCACGCACGAT
468	ATF4	CCCTTCACCTTCTTACAACCTC	TGCCCAGCTCTAAACTAAAGGA
22926	ATF6	AGCAGCACCCAAGACTCAAAC	GCATAAGCGTTGGTACTGTCTGA
3309	BIP	GAACGTCTGATTGGCGATGC	ACCACCTTGAACGGCAAGAA
1649	Chop	GGAAACAGAGTGGTCATTCCC	CTGCTTGAGCCGTTCATTCTC
2730	GCLM	GTGTGATGCCACCAGATTTGAC	CACAATGACCGAATACCGCAGT
3162	HO-1	CAGAGCCTGGAAGACACCCTAA	AAACCACCCCAACCCTGCTAT
2081	IRE1	GAGACCCTGCGCTATCTGAC	CTTGGCCTCTGTCTCCTTGG
4779	Nrf1	GCTGGACACCATCCTGAATC	CCTTCTGCTTCATCTGTCGC
4780	Nrf2	TCAGCGACGGAAAGAGTATGA	CCACTGGTTTCTGACTGGATGT
9451	PERK	CTTCCAGTGGGACCAAGACC	CGAGGTCCGACAGCTCTAAC
5682	PSMA1	ATTCATCAAATTGAATATGCAAT	CTCTGATTGCGCCCTTTTCAA
5685	PSMA4	TTGCTGTACATTGGCTGGGA	ACACAGCTGCAGCGCTATTA
5688	PSMA7	TACATCACCCGCTACATCGC	AGAGCCTAGGAGTGCCATCA
5694	PSMB6	TCAAGAAGGAGGGCAGGTGT	GTAAAGTGGCAACGGCGAA
5695	PSMB7	CTGTGTCGGTGTATGCTCCA	TGCCAGTTTTCCGGACCTTT
5700	PSMC1	ACAAGGTGCATGCCGTGATA	CTGTGCCAGGTGGACCATAG
7494	XBP1	CCCTCCAGAACATCTCCCCAT	ACATGACTGGGTCCAAGTTGT
			

**Table 2:** ARE and AP1-binding sites within -5 kbp to TSS and to TIS of ER-stress gene promoters.

ID	Name	ARE/EpRE (5′-TGAC/GnnnGC-3′)	TRE/AP1 Site (5′-TGAC/GTCA-3′)
3309	BIP/GRP78	TGGCGCAATCTCAGCTC (–4344 to –4328) ATTTTGACCAGGCTGGT (–3811 to –3795) TGGTGCGATCTCAGCTC (–2848 to –2832) TAAGTGACTGTGCTTTG (–2480 to –2464) GAGCTGAGATTGCACTA (–1339 to –1323)	CTCTTGAGTCACCAG (–2104 to –2090) GTACTGAGTCACAGG (–2048 to –2034)
9451	PERK	GGTTTGAGTTCGCTCAT (–2728 to –2712) TCTAGCAAACTCATATA (–1844 to –1828) GCGTGCCAGGTCAGAGT (–719 to –703) CCAATGAGAGAGCAAAC (+60 to +76)	
22926	ATF6	TCTTGCTCTGTCACCCA (–3459 to –3443) GAGCTGAGATGGCTCCA (–2054 to –2038) GTTCTGAGATAGCCACG (–343 to –327)	
1649	Chop	CACAGCTTGGTCATGTC (–4521 to –4505) AAGGGCTACCTCAGTCA (–4384 to –4368) AGGCGCCCTGTCACCCA (–2780 to –2764) TCTCGCTCTGTCACCCA (–935 to –919) AAGCTGAGTTGGCCAGG (+2219 to +2235)	CGCATGACTCACCCA (–242 to –228)
2081	IRE1	ACCCGCCACCTCAGCCT (–4079 to –4063) TGAGTGACTTGGCCGTG (–692 to –676) AGTCTGACGCGGCAGGT (–370 to –354) TGAGGCTCGGTCACCGC (+26 to +42)	
7494	XBP1	TCCCTGACCGAGCTGGT (–4419 to –4403) CACTGCAGCCTCAATCT (–4205 to –4189) CTCAGCCTCCTCAGTAG (–3987 to –3971) ATGTTGACCAGGCTGGT (–3901 to –3885) CTGTTGACCAGGCTGGA (–2943 to –2927) CTGGTGACAGAGCCTGA (–869 to –853) AAATGCACGCTCATAGT (–701 to –685)	GGCATGAGTCACCGT (–4306 to –4292)
468	ATF4	CTGCTGAGATTGCAGTA (–4933 to –4917) ATCTTGAGAGAGCTCAT (–4449 to –4433) ACCATGACTGGGCAAGC (–3612 to –3596) TTGCTGACTGTGCTCCC (–3105 to –3089) GGACTGACTTGGCTGAG (–2940 to –2924) ATTTGCACAGTCATCTG (–2230 to –2214) CCTCTGAGGCAGCAGGA (–1788 to –1773) CCATGCAGACTCAGCCG (–893 to –877)	GGCGTGAGTCAAGGG (+513 to +527)

Note: The red nucleotide letters represent the indicated consensus motifs. TSS and TIS denote the indicated transcriptional start signals and translation initiation signals deciphered in distinct genes, respectively.

## Supplementary Materials

The following are available online at https://www.mdpi.com/2076-3921/9/1/4/s1, Figure S1: A heatmap was made by the Log2-based RPKM values, representing differential expression profiles of those ER stress- responsive genes in *Nrf1α*^−/−^, *Nrf2*^−/−*ΔTA*^, and *caNrf2^ΔN^* cell lines, when compared to those obtained from the wild-type (*Nrf1*^+/+^) cells. Figure S2: Non-significant differences between untreated (i.e., NC) and vehicle (0.1% DMSO) controls used in this study. Figure S3: Analysis of distinct cell apoptosis by flow cytometry as shown graphically. Figure S4: Analysis of the intracellular ROS levels by flow cytometry. Figure S5: The mean DCFH-FITC values and their fold changes of all the examined cells. Additional data related to this paper may also be requested from the authors.
