# Supplementary material for: Unification of Opposites between Two Antioxidant Transcription Factors Nrf1 and Nrf2 in Mediating Distinct Cellular Responses to the Endoplasmic Reticulum Stressor Tunicamycin
Source: Antioxidants (Basel). 2019 Dec 19;9(1):4. doi: 10.3390/antiox9010004 (PMC7022656; doi:10.3390/antiox9010004)
Supplement: Supplementary file 1 [file antioxidants-09-00004-s001.pdf]

Unification of opposites between two antioxidant transcription factors Nrf1 and Nrf2 in mediating distinct cellular responses to the endoplasmic reticulum stressor tunicamycin

Yu-ping Zhu[1,§], Ze Zheng[1,§], Shaofan Hu[1,§], Xufang Ru[1,2], Zhuo Fan[1], Lu Qiu[1,3] and Yiguo Zhang[1,*]

[1]The Laboratory of Cell Biochemistry and Topogenetic Regulation, College of Bioengineering, Chongqing University, No. 174 Shazheng Street, Shapingba District, Chongqing 400044, China.[2] Department of Neurosurgery, Southwest Hospital, Army (Third Military) Medical University, No. 29 Gaotanyan Street, Shapingba District, Chongqing 400038, China.[3]School of Life Sciences, Zhengzhou University, No. 100 Kexue Avenue, Zhengzhou 450001, Henan, China.
[§]Contributed equally to this work.
*****Correspondence: yiguozhang@cqu.edu.cn; eaglezhang64@gmail.com

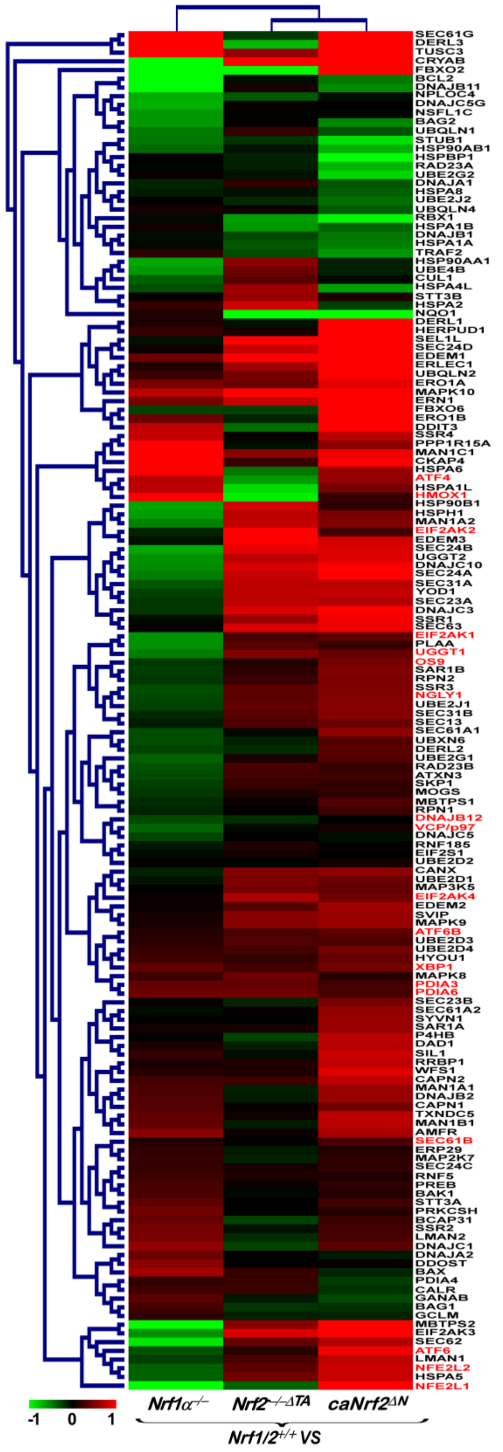

Supplementary Figure S1. A heatmap was made by the Log2-based RPKM values, representing differential expression profiles of those ER stress-responsive genes in *Nrf1α*$^{-/-}$, *Nrf2* $^{-/-\Delta TA}$, and *caNrf2*$^{\Delta N}$ cell lines, when each of which compared to those obtained from the wild-type (*Nrf1*$^{+/+}$) cells. Changes in the basal expression of these genes are shown, to distinct degrees of colours. Of note, this is an enlarged image with a higher resolution, that was also shown in the main Figure 2B.

Supplemental Figure S2

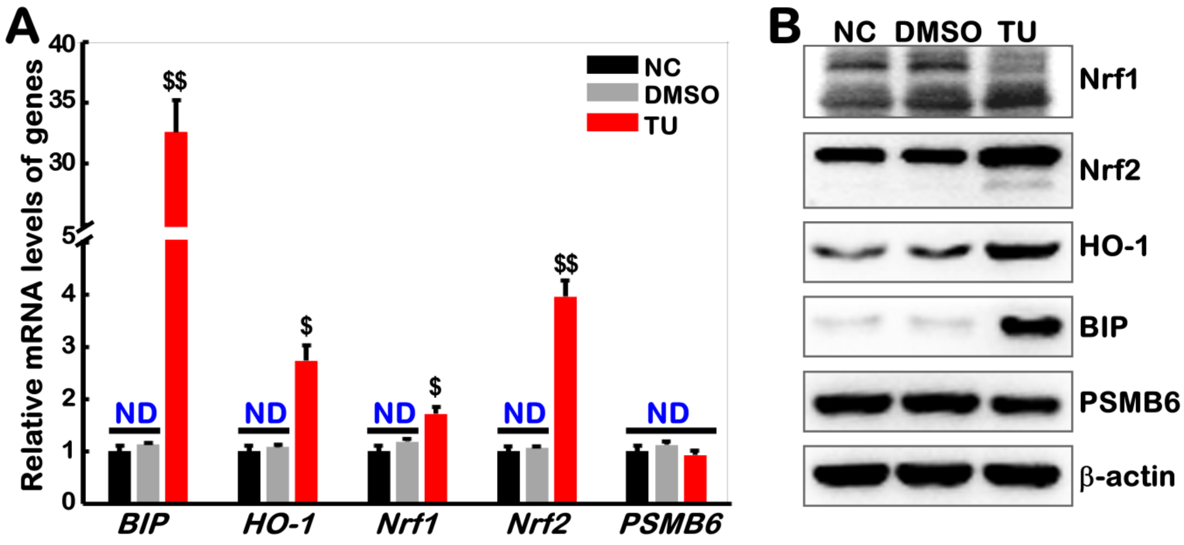

Supplementary Figure S2. There are non-significant differences between untreated (i.e., NC) and vehicle (0.1% DMSO) controls used in this study. These repeatable experiments have been carried out to validate the non-significant differences between untreated (NC) and 0.1% DMSO vehicle controls. The results of qRT-PCR analysis (in *Panel A*) showed no significant differences (ND) between untreated (NC) and 0.1% DMSO vehicle treated mRNA expression levels of *BIP*, *HO-1*, *Nrf1*, *Nrf2,* and *PSMB6* genes in HepG2 cells, which had been untreated or treated with 0.1% DMSO for 12 h. By contrast, treatment of cells with TU (2 μg/ml resolved in DMSO) caused significant increases in the transcriptional expression of *BIP*, *HO-1*, *Nrf1*, *Nrf2*, but not *PSMB6*. Furtherly, almost no changes in basal expression levels of Nrf1, Nrf2, BIP, HO-1 and PSMB6 proteins in HepG2 cells, that had been untreated (NC) and treated with 0.1% DMSO for 12 h, were determined by Western blotting (in *Panel* B), when compared with the corresponding positive stimulation by TU (2 μg/ml). These supportive data are a representative of at least three independent experiments (n=9). Significant differences in gene expression were subjected to statistical analysis. Significant increases ($, p<0.05; $$, p<0.01), along with non-significant differences (ND), are shown.

Supplemental Figure S3

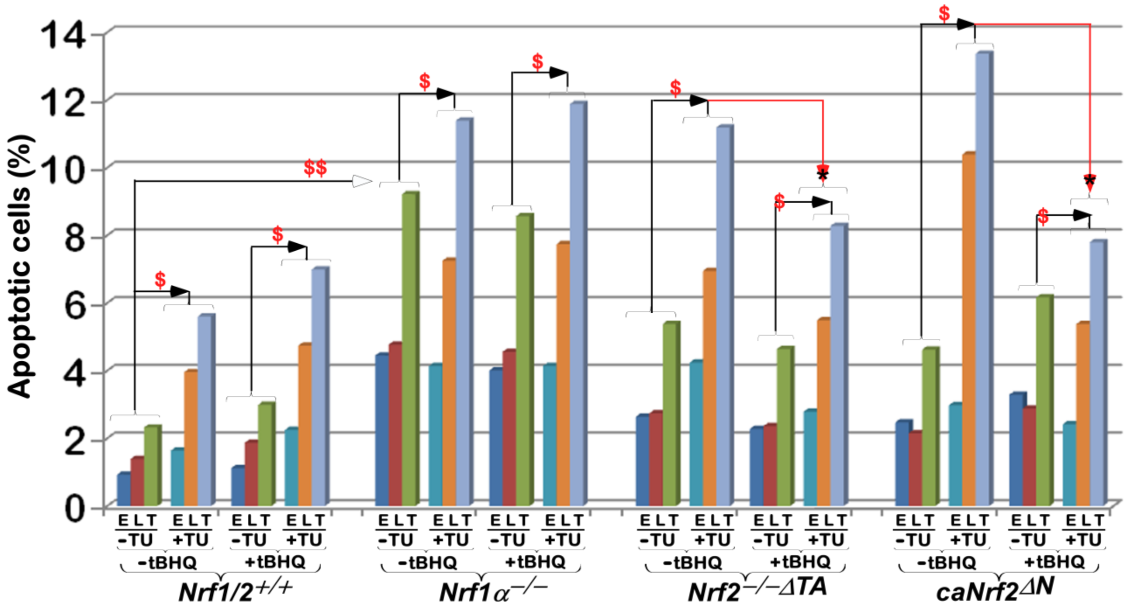

Supplementary Figure S3. Cell apoptosis by flow cytometry analysis are here shown graphically (E, early apoptosis; L, late apoptosis; T, total apoptosis). Four different cell lines *Nrf1/2*$^{+/+}$, *Nrf1α*$^{-/-}$, *Nrf2*$^{-/-\Delta TA}$, and *caNrf2*$^{\Delta N}$ were pretreated with 50 μmol/L tBHQ or 0.1% DMSO vehicle (i.e., –tBHQ) for 16 h, before they were or were not treated with 2 μg/ml of TU for additional 48 h. Subsequently, these cells were incubated in a binding buffer containing both Annexin V-FITC and propidium iodide for 15 min, before being subjected to flow cytometry analysis of these cell apoptosis. The resulting data were then analyzed by the FlowJo 7.6.1 software, and calculated as a mean percent change (n=3), which represent at least three independent experiments. Significant differences in cell apoptosis were also subjected to statistical analysis. Significant increases ($, *p*<0.05) and significant decreases (**p*<0.05) are shown. Of note, relevant data were shown in the main Figure 7B.

Supplemental Figure S4

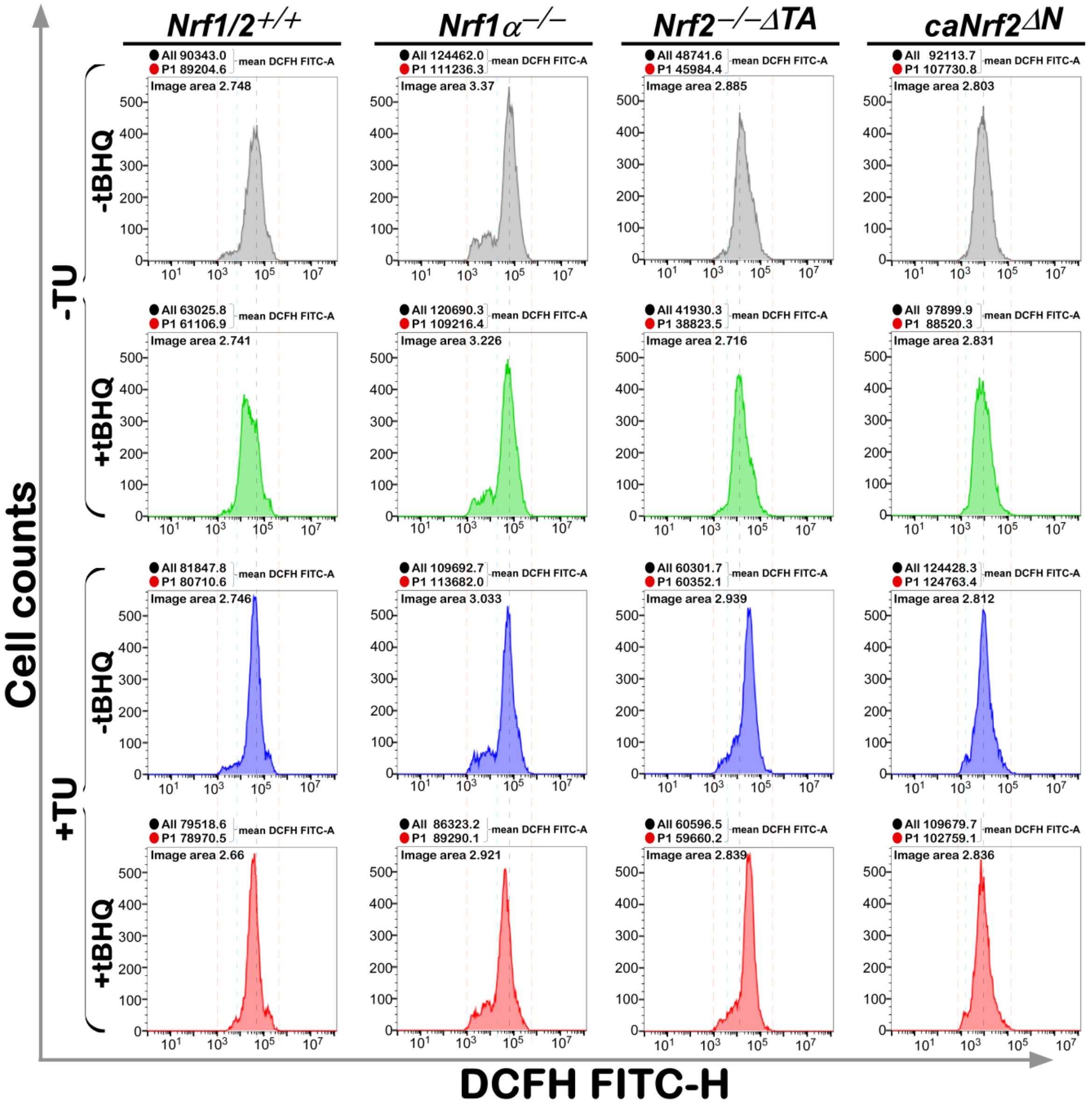

Supplementary Figure S4. The intracellular ROS levels by flow cytometry analysis are illustrated. Experimental cells of *Nrf1/2*$^{+/+}$, *Nrf1α*$^{-/-}$, *Nrf2*$^{-/-ΔTA}$ and *caNrf2*^{ΔN} were allowed for growth in 6-well plates. After reaching 70% of their confluence, the cells were transferred in fresh media containing tBHQ (50 μmol/L) or the vehicle DMSO (i.e., –tBHQ) for 16 h, before they were or were not treated with 2 μg/ml of TU for additional 48 h. Subsequently, the experimental cells were incubated for 20 min at 37℃ in a serum-free medium containing 10 μmol/L of 2′,7′-dichlorodihydrofluorescein diacetate (DCFH-DA) to detect the intracellular H_2O_2 levels . The staining cells were rinsed for three times in a serum-free medium, followed by flow cytometry analysis of the intracellular green fluorescent intensity (representing the ROS levels). The mean DCFH-FITC values of all the examined cells or P1 grouped cells were shown on the top of each box. In addition, each image area was also estimated by the ImageJ software. Of note, the overlapped images obtained from each cell line were also shown in the main Figure 8A.

Supplemental Figure S5

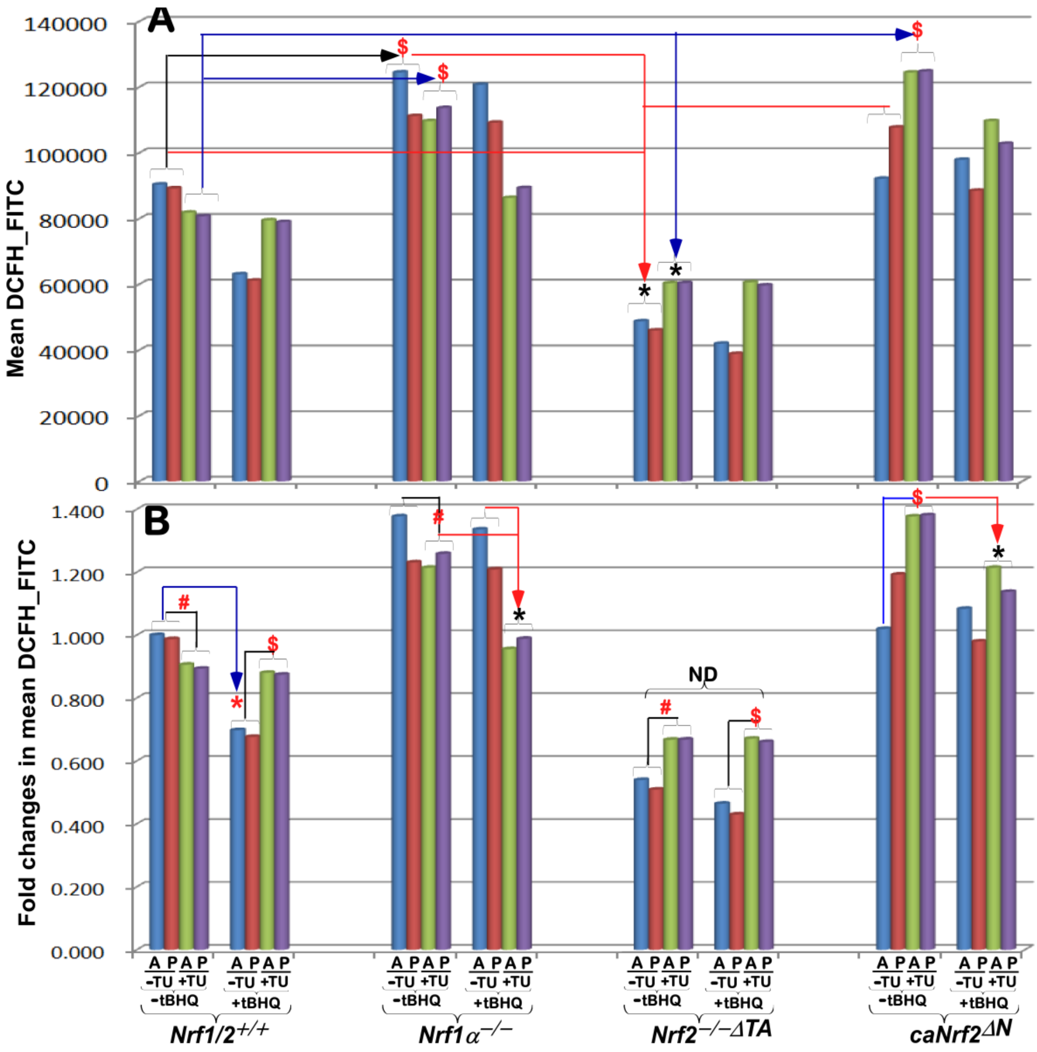

Supplementary Figure S5. The A *panel* shows that the mean DCFH-FITC values of all the examined cells (i.e., *A*) or P1 grouped cells (i.e., *P*) were obtained from flow cytometry analysis of these intracellular ROS levels. The B *panel* shows that all the obtained values were then normalized to that of all examined *Nrf1/2*$^{+/+}$ cells (at a given value of 1). The resulting data were then analyzed by the FlowJo 7.6.1 software, and calculated as a mean percent change (n=3), which represent at least three independent experiments. Significant differences in the mean intracellular ROS levels were subjected to statistical analysis. Significant increases ($, p<0.05) and significant decreases (*p<0.05) are shown, but no significant difference is represented by the symbol (#), beside ND = no differences. Of note, for detailed description, please see the legends of Figures 8A and S4.
